# Oxidative Stress and *SIRT1*-Nrf2 Anti-Ferroptotic Pathways in Granulosa Cells: A Molecular Key to Follicular Atresia and Ovarian Aging

**DOI:** 10.3390/ijms27020950

**Published:** 2026-01-18

**Authors:** Charalampos Voros, Fotios Chatzinikolaou, Georgios Papadimas, Spyridon Polykalas, Despoina Mavrogianni, Aristotelis-Marios Koulakmanidis, Diamantis Athanasiou, Vasiliki Kanaka, Kyriakos Bananis, Antonia Athanasiou, Aikaterini Athanasiou, Ioannis Papapanagiotou, Charalampos Tsimpoukelis, Athanasios Karpouzos, Maria Anastasia Daskalaki, Nikolaos Kanakas, Marianna Theodora, Nikolaos Thomakos, Panagiotis Antsaklis, Dimitrios Loutradis, Georgios Daskalakis

**Affiliations:** 1 1st Department of Obstetrics and Gynecology, ‘Alexandra’ General Hospital, National and Kapodistrian University of Athens, 80 Vasilissis Sofias Avenue, 11528 Athens, Greece; 2 Laboratory of Forensic Medicine and Toxicology, School of Medicine, Aristotle University of Thessaloniki, 54124 Athens, Greece; fotischatzin@auth.gr (F.C.);; 3 Athens Medical School, National and Kapodistrian University of Athens, 15772 Athens, Greece; 4 King’s College Hospitals NHS Foundation Trust, London SE5 9RS, UK; 5 IVF Athens Reproduction Center, 15123 Maroussi, Greece; 6 School of Medicine, European University Cyprus, Nicosia 2404, Cyprus; 7 Fertility Institute-Assisted Reproduction Unit, Paster 15, 11528 Athens, Greece

**Keywords:** infertility, oxidative stress, *SIRT1*, Nrf2, ferroptosis, ovarian aging, antioxidants, IVF, reproductive endocrinology, molecular research

## Abstract

The functional deterioration of granulosa cells (GCs), essential for follicular growth, steroidogenesis, and oocyte competence, indicates ovarian aging and reduced fertility. An expanding corpus of research indicates that oxidative stress is a primary molecular contributor to granulosa cell dysfunction, culminating in mitochondrial impairment, reduced metabolic support for oocytes, and the activation of regulated apoptotic pathways that end in follicular atresia. Ferroptosis, an emergent type of iron-dependent lipid peroxidation, has been identified as a crucial mechanism contributing to chemotherapy-induced ovarian insufficiency, polycystic ovary syndrome (PCOS), and granulosa cell death in aging ovaries, in addition to conventional apoptosis. The *SIRT1*-*Nrf2* axis acts as a crucial anti-oxidative and anti-ferroptotic system that protects GC viability, maintains mitochondrial homeostasis, and upholds redox equilibrium. *SIRT1* promotes mitochondrial biogenesis and metabolic resilience by deacetylating downstream proteins, including FOXO3 and PGC-1α. Nrf2 simultaneously controls the transcriptional activation of detoxifying and antioxidant enzymes, including HO-1, SOD2, NQO1, and GPX4, which are critical inhibitors of ferroptosis. Disruption of *SIRT1*-Nrf2 signalling accelerates GC senescence, follicular depletion, and reproductive aging. In contrast, pharmaceutical and nutraceutical therapies, including metformin, melatonin, resveratrol, and agents that increase NAD^+^ levels, may reverse ovarian deterioration and reactivate *SIRT1*-Nrf2 activity. This narrative review highlights innovative treatment prospects for ovarian aging, fertility preservation, and assisted reproduction by synthesising current evidence on ferroptotic pathways, *SIRT1*-Nrf2 interactions, and oxidative stress in granulosa cells. An understanding of these interrelated biological networks enables the development of tailored therapies that postpone ovarian ageing and enhance reproductive outcomes for women receiving fertility therapy.

## 1. Introduction

### 1.1. Granulosa Cells as the Metabolic and Regulatory CORE of the Follicle

The specialised somatic network of GCs establishes the biochemical, metabolic, endocrine, and epigenetic milieu essential for oocyte growth and maturation. Granulosa cells are not only passive support cells [1]. They function as active regulatory centres that transmit signals between cells, react to endocrine signals from gonadotropins, and provide metabolic and paracrine directives to the developing oocyte. Gap junctions abundant in connexin-43 and dynamic transzonal projections across the zona pellucida facilitate communication between granulosa cells and oocytes [2]. These function as conduits for the transport of ions (Ca^2+^), metabolites (pyruvate, lactate), amino acids, nucleotides, and regulatory macromolecules such as microRNAs, mitochondrial signals, and cAMP. This ensures that oocyte nuclear maturation and cytoplasmic remodelling occur simultaneously [3]. The oocyte serves as the primary source of guidance by secreting growth differentiation factor-9 (GDF9) and bone morphogenetic protein-15 (BMP15). These chemicals stimulate SMAD-dependent pathways in granulosa cells to facilitate proliferation, preserve cumulus identity, inhibit premature luteinisation, and modify the expression of the FSH receptor (FSHR), hence augmenting the follicular response to gonadotropins.

FSH stimulation initiates a cAMP–PKA cascade in granulosa cells that regulates the activity of AMP-activated protein kinase (AMPK), AKT, and mTOR, while also promoting the transcription of steroidogenic enzymes such as StAR, CYP11A1, and CYP19A1 (aromatase). This is executed to synchronise metabolism and growth [4]. GCs regulate substrate utilisation, guide glycolytic flow to produce pyruvate (the preferred energy source for oocytes), and oversee mitochondrial biogenesis and redox equilibrium to protect the oocyte from metabolic stress. The metabolic dependency of the follicular unit is highlighted by the intrinsic metabolic constraints of oocytes, which depend on granulosa cells to convert glucose into ATP precursors and NAD^+^ intermediates [5].

At the cellular level, GCs possess a highly dynamic mitochondrial network. Mitochondrial integrity, membrane potential (ΔΨm), ATP synthesis, and lipid metabolism directly influence oestradiol generation, follicular growth, and oocyte functionality [6]. The mitochondrial-associated endoplasmic reticulum membranes in germ cells regulate calcium homeostasis, facilitate cholesterol transport for steroidogenesis, and modulate mitochondrial fission and fusion dynamics. Any impairment to this mitochondrial structure, whether due to oxidative stress, gonadotoxic agents, or aging, may inhibit steroidogenesis, ATP synthesis, and oocyte development [7].

Granulosa cells are the endocrine regulators of follicular maturation since they produce oestradiol, progesterone, inhibin-B, activin, and AMH. They regulate follicular recruitment and feedback from the hypothalamus and pituitary gland [8]. As the follicle develops, its secretory phenotype undergoes dynamic alterations, resulting in modifications to tight junction remodelling, cytoskeletal reorganisation, and LH-dependent luteinisation processes that facilitate follicular growth and preparation for ovulation. GCs affect transcriptional reprogramming and meiotic development by modifying histone acetylation, regulating chromatin via sirtuins, and exchanging long non-coding RNAs with the oocyte at the epigenetic level [9]. The quality of the oocyte is ultimately contingent upon the health of the granulosa cells. Structural or metabolic impairment of the GC results in diminished cumulus growth, inadequate support for spindle formation, mitochondrial dysfunction in oocytes, aneuploidy, meiotic failure, and follicular atresia due to apoptosis or ferroptosis. Consequently, granulosa cells serve as the paramount guardians of ovarian reserve, endocrine functionality, and reproductive efficacy. They constitute not only a collection of supportive cells; rather, they form a pivotal regulatory axis that governs the duration and quantity of a woman’s reproductive capacity [10].

### 1.2. Oxidative Stress Is the Primary Determinant in Follicular Atresia and Ovarian Senescence

**Figure 1:**
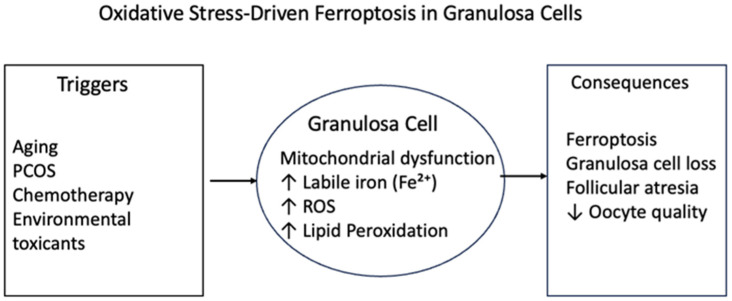
Oxidative stress induces ferroptosis in granulosa cells. Chronic oxidative stress resulting from ageing, metabolic disorders, chemotherapy, or environmental toxins may disrupt the iron equilibrium and mitochondrial activity of granulosa cells. Ferroptotic cell death occurs when ROS accumulate, the labile iron pool increases, and lipid peroxidation transpires. This results in the demise of granulosa cells, follicular atresia, and suboptimal oocyte quality.

These events include mitochondrial malfunction, metabolic failure, and regulated apoptosis [11]. In the ovarian microenvironment, ROS are generated as byproducts of oxidative phosphorylation within the electron transport chain, notably at complexes I (NADH dehydrogenase) and III (cytochrome bc1 complex), where superoxide anions (O_2_^•−^) result from electron leakage [12]. GCs maintain a meticulously controlled redox milieu in young and metabolically stable states via a network of antioxidant defences, including GPX4, catalase, SOD2, and the thioredoxin/thioredoxin reductase system. They use effective mitochondrial quality-control mechanisms, including PINK1-Parkin-dependent mitophagy and sirtuin-regulated mitochondrial biogenesis [13]. A fundamental mechanism linking iron dysregulation, follicular atresia, and mitochondrial dysfunction is the progressive transition from oxidative stress to ferroptotic death of granulosa cells (Figure 1).

The decline of the mitochondrial NAD^+^ pool due to ovarian aging, hormonal stress, environmental damage, or gonadotoxic exposure impacts the NAD^+^/NADH ratio, diminishes *SIRT1* and *SIRT3* activity, and promotes a metabolic transition from oxidative phosphorylation to anaerobic glycolysis [14]. The decrease in sirtuin-mediated deacetylation hinders PGC-1α-induced mitochondrial biogenesis. This leads to decreased transcription of mitochondrial respiratory components, impaired replication of mtDNA, and changes in mitochondrial morphology, such as fragmentation, cristae disruption, and loss of membrane potential (ΔΨm). Impaired electron transport elevates superoxide generation and hydrogen peroxide buildup, depletes glutathione stores, and reduces the GSH/GSSG ratio, which is crucial for sustaining redox homeostasis [15,16]. In granulosa-cell subpopulations subjected to chronic inflammatory signalling, oxidative stress simultaneously induces caspase-1 activation, maturation of IL-1β/IL-18, and pyroptotic tendencies via activating NF-κB through IκB degradation and facilitating NLRP3 inflammasome assembly. Inhibition of AKT and mTOR signalling limits protein synthesis, hinders autophagic flux, and diminishes cellular survival ability. The JNK and p38 MAPK pathways concurrently exacerbate oxidative damage by phosphorylating pro-apoptotic proteins and regulating the transcription of antioxidant genes [17].

Senescent GCs have a senescence-associated SASP marked by the secretion of inflammatory cytokines, matrix-degrading enzymes, and reactive aldehydes [18]. This trait establishes a self-replicating pro-oxidative milieu that propagates damage to adjacent cells and accelerates follicular degeneration. Mitochondrial stress and ER stress are tightly linked at the organelle level via the PERK, IRE1α, and ATF6 pathways. This leads to the activation of the UPR. The continuous activation of the UPR enhances CHOP-mediated apoptotic signalling, suppresses oestradiol production, and disrupts the folding of steroidogenic enzymes [19]. MAMs are essential for cholesterol transport and calcium exchange; nevertheless, their structural integrity declines, impairing steroidogenesis and mitochondrial function. The capacity of granulosa cells to facilitate oocyte maturation is eventually undermined by simultaneous deficiencies in mitochondrial function, redox equilibrium, endoplasmic reticulum integrity, and DNA repair mechanisms [20]. This leads to meiotic arrest, spindle instability, chromosomal missegregation, and developmental incompetence of the egg. Oxidative stress is a primary factor that dictates the molecular process of granulosa-cell failure, rather than a mere consequence of aging. Oxidative stress alters mitochondrial bioenergetics, destroys genomic and mitochondrial DNA, triggers inflammatory and apoptotic pathways, and imposes senescence processes, building the molecular basis for ferroptosis sensitisation, metabolic failure, and follicular atresia. To sustain female fertility, delay reproductive aging, and conserve ovarian reserve, it is essential to protect granulosa cells from oxidative harm [21].

This study first elucidates the biological function of granulosa cells as the metabolic and redox-regulatory hub of the ovarian follicle. Subsequently, we discuss the gradual impairment of their endocrine and mitochondrial integrity due to oxidative stress. We highlight iron dysregulation, lipid peroxidation, and the dysfunction of the glutathione–GPX4 axis as key causes of follicular atresia, concentrating on ferroptosis as a unique and important kind of controlled cell death in granulosa cells. Subsequently, the protective functions of the *SIRT1* and Nrf2 signalling pathways in preserving redox equilibrium, mitochondrial integrity, and resistance to ferroptotic damage are examined. Ultimately, we discuss the implications of these molecular findings on oocyte quality, IVF success, ovarian ageing, and novel therapeutic approaches that integrate clinical and translational data.

Previous studies have thoroughly investigated oxidative stress and ovarian ageing, primarily concentrating on mitochondrial malfunction, apoptosis, autophagy, or a general imbalance of antioxidants. This study finds iron dysregulation and lipid peroxidation as the primary causes of follicular atresia, specifically including ferroptosis as a separate, mechanistically controlled, and highly relevant type of granulosa cell death. This study highlights the *SIRT1*-Nrf2 axis as a collaborative anti-ferroptotic and pro-survival signalling network that encompasses metabolic sensing, mitochondrial quality control, and antioxidant defence, rather than concentrating just on redox regulation. By combining these pathways, we want to provide a mechanistic framework that surpasses simplistic descriptive models of oxidative stress, yielding new insights into follicular depletion and ovarian ageing.

Our evaluation establishes crucial terminology initially and use them regularly for clarity. Oxidative stress is an unfavourable disparity between the generation of reactive oxygen species and the body’s capacity to neutralise them. It adversely affects proteins, lipids, and nucleic acids. Apoptosis, a regulated form of cell death reliant on caspases and characterised by the absence of cell lysis, is distinguished by DNA fragmentation and the permeabilization of the mitochondrial outer membrane. Autophagy, a lysosome-mediated recycling mechanism, may exert either cytoprotective or cell death associated effects, and is contingent upon the context. Necroptosis is a controlled necrotic mechanism triggered by membrane rupture and RIPK1/RIPK3-MLKL signalling. This study primarily examines ferroptosis, a unique iron-dependent kind of controlled cell death that differs from conventional cell death modes in both shape and mechanism. Phospholipid peroxidation, glutathione depletion, and GPX4 inactivation are the causative factors.

## 2. Methods

Our narrative review aimed to integrate experimental, translational, and mechanistic findings about ferroptosis, oxidative stress, and the *SIRT1*-Nrf2 regulatory axis in granulosa cell biology and ovarian aging. The literature search method was deliberately broad and iterative to include the exploratory dimensions of emerging pathways in ovarian redox signalling and controlled cell death. The principal databases used were PubMed/MEDLINE and Scopus, augmented by further examination via Web of Science and Google Scholar to guarantee the incorporation of contemporary reviews, pioneering biochemical research, and significant experimental articles. The search terms included combinations of “granulosa cells,” “oxidative stress,” “mitochondria,” “ferroptosis,” “GPX4,” “SLC7A11,” “iron metabolism,” “*SIRT1*,” “Nrf2,” “oocyte competence,” “follicular atresia,” and “ovarian aging,” as well as related molecular modifiers such as “NCOA4,” “ACSL4,” “FOXO3,” “PGC-1α,” “mitophagy,” and “NAD^+^ metabolism.”

We concentrated on research using primary human granulosa cells, cumulus cells from IVF cycles, animal ovarian models, and in vitro granulosa cell lines (such as KGN and COV434) to elucidate the unique cellular milieu critical for folliculogenesis. We favoured experimental research that provided mechanistic insights into ovarian cell mitochondrial stress signalling, redox homeostasis, and lipid peroxidation pathways, even if conducted in non-reproductive cell systems, provided the pathways were directly pertinent to granulosa cell physiology. Only high-quality mechanistic evaluations that provided critical conceptual frameworks or historical molecular insights were considered.

The publications were evaluated for their scientific quality, topical relevance, and translational importance in reproductive medicine. Research concentrating only on apoptosis or necrosis, without accounting for oxidative or ferroptotic dynamics, was deprioritized unless it showed significant mechanistic connections to upstream mitochondrial or iron-regulatory mechanisms. Although the emphasis was on material from the last 10 to 12 years to illustrate the rapid evolution of ferroptosis, sirtuin, and redox biology, there were no restrictions on publication dates or languages. The reference lists of important articles were thoroughly examined to locate other relevant sources.

This narrative review did not use a rigorous meta-analytic methodology, bias-assessment methods, or a study-selection diagram. The data was qualitatively synthesised, highlighting biological plausibility, pathway integration, molecular coherence, and mechanistic relevance to aging and ovarian physiology. Discrepancies found in the literature were meticulously assessed based on the model system, cell type, experimental methodology, and consistency with current redox and ferroptosis biology. This methodological approach aimed to incorporate innovative mechanistic discoveries into a unified biological narrative that aligns with the contemporary knowledge at the convergence of controlled cell-death signalling, mitochondrial medicine, and reproductive endocrinology.

## 3. Granulosa Cell Oxidative Stress: Molecular Aetiology, Experimental Results, and Clinical Implications

In GCs, oxidative stress is the primary molecular perturbation that progressively destabilises mitochondrial dynamics, alters steroidogenic potential, disrupts RNA and protein quality control, and ultimately leads to follicular atresia. In reproductive physiology, oxidative signals are not invariably detrimental [22]. Cyclic fluctuations in ROS affect follicle recruitment, steroidogenesis, and ovulation. But pathological ROS buildup overloads the body’s natural detoxification systems, which starts the process of going from adaptive signalling to permanent cell damage.

### 3.1. Sources and Biochemical Drivers of Oxidative Stress in Granulosa Cells

This is particularly accurate when the cells exhibit heightened activity, such as during follicular development or steroidogenesis. Electrons escaping from complexes I (NADH dehydrogenase) and III (cytochrome bc1 complex) in the electron transport chain result in the formation of superoxide radicals [23]. Typically, these radicals are neutralised by mitochondrial antioxidant mechanisms. Due to mitochondrial malfunction associated with reproductive aging, intracellular NAD^+^ levels are diminished, *SIRT1* and *SIRT3* activity is reduced, and PGC-1α’s capacity to stimulate mitochondrial biogenesis is also diminished [24]. Mitochondria with structural impairments exhibit depolarised membranes, impaired cristae, diminished ATP synthesis, and very inefficient electron transport. These issues accumulate progressively. When these impaired mitochondria become persistent generators of superoxide and hydrogen peroxide, they establish a self-sustaining cycle of redox degradation and energy imbalance [25].

The regulation of mitochondrial fusion and fission also influences the production of ROS. Proteins such as OPA1 and MFN1/2 maintain the integrity of extensive, linked mitochondrial networks by enhancing electron transport efficiency and preventing the leakage of ROS [26]. Oxidative stress causes mitochondria to fragment into smaller organelles that exhibit impaired bioenergetic function and produce excessive ROS. The alteration in mitochondrial morphology is both a causative factor and a consequence of oxidative damage. It links redox instability with bioenergetic dysfunction. Oxidative lesions induced by ROS are particularly detrimental to mtDNA since they impair the respiratory chain’s ability to synthesise subunits, hence compromising mitochondrial function [27].

Granulosa cells generate ROS via NADPH oxidases, particularly NOX4, which is activated by hormonal stress, elevated testosterone levels, metabolic disorders, and inflammatory cytokines such as TNF-α and IL-1β. ROS from NOX4 accelerate cellular apoptosis by elevating mitochondrial ROS levels and obstructing redox-sensitive transcription factors [28]. Disruptions in endoplasmic reticulum-mitochondria communication via MAMs may lead to excessive Ca^2+^ release from the endoplasmic reticulum and excessive Ca^2+^ uptake by the mitochondria. This activates the unfolded protein response (UPR^ER^) and the synthesis of ROS [29]. To re-establish equilibrium, misfolded proteins activate PERK, IRE1α, and ATF6. Nonetheless, prolonged or excessive activation of UPR inhibits aromatase maturation, diminishes oestradiol production, initiates CHOP-mediated apoptotic pathways, and elevates oxidative stress.

As oxidative stress increases, ROS directly oxidise polyunsaturated phospholipids in mitochondrial and cellular membranes, impair GSH redox buffering, and compromise the antioxidant systems of thioredoxin and peroxiredoxin [30]. The magnitude and duration of oxidative stress determine the ensuing death pathways: Significant depletion of GSH and unregulated lipid peroxidation cause granulosa cells to gradually undergo ferroptosis, while moderate ROS levels trigger caspase-dependent apoptosis through the permeabilization of the mitochondrial outer membrane, connecting oxidative damage to iron-dependent lipid-damage pathways that are essential in follicular atresia.

### 3.2. Antioxidant Defense Networks and Their Dysregulation in Ovarian Aging

The complex antioxidant defence mechanism of granulosa cells safeguards the follicular milieu and oocyte from oxidative damage, maintaining the endocrine integrity of the ovary and the developmental potential of the oocyte [31]. This antioxidative system functions well under normal circumstances, safeguarding cells against fluctuations in ROS that occur during luteinisation, cellular respiration, and steroidogenesis. The defence network comprises metabolic pathways that maintain steady glutathione levels, enzymatic antioxidants, redox-regulating transcription factors, and mitochondrial quality control mechanisms. All these elements collaborate to maintain redox equilibrium in granulosa cells [32].

Numerous downstream enzymes, including GPX4, a selenoenzyme that inhibits ferroptosis) and catalase (which decomposes hydrogen peroxide into water and oxygen), are subsequently used to eliminate this intermediate oxidant. GPX4’s capacity to reduce phospholipid hydroperoxides in mitochondrial and plasma membranes is crucial for the viability of granulosa cells [33]. This is due to its inhibition of lipid-peroxidation chain events that may otherwise harm membranes, alter the structure of mitochondrial cristae, and initiate ferroptosis. The production and recycling of GSH constitute a crucial component of the antioxidative defence in granulosa cells. GSH stabilises protein thiols and facilitates the elimination of hydrogen and lipid peroxides. The synthesis requires glycine, glutamate, and cysteine, with the availability of cysteine being the limiting factor in the process. The Xc^−^ transporter system (SLC7A11/SLC3A2) is the primary mechanism by which granulosa cells import cysteine in exchange for glutamate [34]. Upon entering the cell, glutamate-cysteine ligase (GCLC and GCLM) converts cystine into cysteine and incorporates it into GSH. Antioxidant signalling emphasises this whole axis since Nrf2 regulates it at the transcriptional level.

The Nrf2-Keap1-ARE pathway is the primary mechanism by which granulosa cells acclimatise to oxidative stress. Nrf2 is bound to Keap1 and is subject to proteasomal degradation when inactive. Oxidative stimuli induce the oxidation of Keap1’s cysteine residues, therefore liberating Nrf2 and facilitating its translocation into the nucleus [35]. Upon entering the nucleus, Nrf2 binds to AREs and promotes the transcription of many antioxidant genes, including ferritin heavy-chain (FTH1), HO-1, NQO1, GCLC/GCLM, thioredoxin reductase, SOD2, and GPX4. This transcriptional response restores redox equilibrium, detoxifies electrophiles, facilitates iron retention within cells, and fortifies mitochondrial membranes against lipid peroxidation. Nrf2 functions as a crucial anti-ferroptotic barrier in the follicle, with multiple genes directly opposing ferroptotic stimuli [36].

The antioxidant capacity of granulosa cells is profoundly affected by mitochondrial quality-control processes, especially mitophagy. The PINK1/Parkin system inhibits the formation of organelles that generate excessive ROS, release cytochrome c, and leak oxidised lipids into the cytoplasm by selectively identifying and eliminating dysfunctional mitochondria [37]. Mitochondrial fission and fusion proteins, including MFN1, MFN2, OPA1, and DRP1, alter mitochondrial morphology to enhance energy production efficiency and reduce ROS generation. Effective mitochondrial turnover maintains ATP synthesis, augments steroidogenic enzyme activity, and delays the premature activation of ferroptotic and apoptotic signals emanating from the mitochondria [38].

With advancing ovarian age, the robust antioxidant defence mechanism becomes significantly dysregulated at the transcriptional, physiological, metabolic, and epigenetic levels. Decreased NAD^+^ concentrations result in diminished *SIRT1* and *SIRT3* activity, impairments in mitochondrial biogenesis, reduced mitophagic flux, and a compromised capacity of cells to respond to oxidative stress [39]. Furthermore, diminished sirtuin activity impedes the deacetylation process of FOXO transcription factors, hence obstructing their ability to activate repair pathways and antioxidant enzymes. Consequently, granulosa cells experience mitochondrial dysfunction and have less capacity to combat ROS [40]. The Nrf2 pathway diminishes in efficacy with advancing age. ARE-driven transcription decreases, resulting in less Nrf2 nuclear translocation and increased expression of Keap1. This results in diminished GPX4 levels, insufficient storage of redox-active iron, and inadequate synthesis of GSH. Under conditions of diminished antioxidant levels, granulosa cells are very susceptible to lipid peroxidation, particularly when subjected to gonadotoxic drugs, metabolic stresses, or inflammatory cytokines [41].

Deficiencies in mitochondrial dynamics and mitophagy exacerbate oxidative instability. With advancing age, dysfunctional mitochondria accumulate, while levels of PINK1 and Parkin diminish, and DRP1-mediated fission increases. This results in highly fragmented mitochondrial networks that may produce excessive ROS [42]. Dysfunctional mitochondria not only generate increased ROS but also release oxidised lipids and mitochondrial DNA, hence activating innate immune pathways such as cGAS-STING and intensifying inflammatory damage inside the follicle. Granulosa cells exposed to chronic oxidative stress have a senescence-associated SASP, marked by the release of chemokines, matrix-degrading enzymes, prostaglandins, inflammatory cytokines (IL-6, TNF-α), and metabolites that increase reactive oxygen species [39]. The SASP environment significantly impedes communication between granulosa cells and the oocyte, undermines the metabolic and structural support essential for oocyte maturation, and propagates oxidative damage to adjacent cells. This finally results in a self-perpetuating redox-inflammatory cycle that accelerates the shift from functional granulosa cells to senescent, defective cells incapable of supporting follicular growth [43]. As antioxidant defence mechanisms deteriorate with ovarian aging, granulosa cells transition from effective guardians of oocyte health to cells more susceptible to ferroptosis and oxidative stress-induced mortality. This alteration signifies a significant juncture in ovarian decline and provides a robust mechanistic rationale for the deterioration of egg quality, age-related infertility, and diminished ovarian reserve.

### 3.3. Clinical Translation: Oxidative Stress, Oocyte Quality, and IVF Outcomes

Prior to discussing the clinical implications, it is crucial to recognise that, notwithstanding the scarcity of direct human clinical data, a significant amount of the molecular information in this review originates from animal research and in vitro granulosa-cell models. Thus, the next part integrates experimental, translational, and clinical data, clearly differentiating between evidence obtained from human IVF situations and preclinical findings.

Examining the direct and quantifiable effects of oxidative stress on oocyte competence, embryo development, implantation potential, and overall IVF success highlights the clinical significance of this situation in granulosa cells [44]. Oxidative instability in the follicular microenvironment adversely impacts meiotic spindle integrity, chromosome segregation accuracy, nuclear and cytoplasmic maturation of the oocyte, mitochondrial inheritance patterns, and early embryonic developmental pathways, while also undermining the metabolic and paracrine functions of granulosa cells [45].

Granulosa cells serve as the primary metabolic regulators in the ovaries. They regulate the redox conditions experienced by the oocyte. Despite the oocyte’s limited antioxidant capacity, it remains susceptible to peroxidised lipids, oxidised proteins, and DNA-damaging radicals when granulosa cells exhibit elevated levels of ROS [46]. Oxidative damage develops in the nuclear and mitochondrial DNA of oocytes, diminishing ATP generation, impairing spindle assembly checkpoint function, and elevating the chance of aneuploidy. Oxidative stress impedes the progression from the germinal vesicle stage to metaphase II, leading to clinically evident abnormalities marked by diminished oocyte maturation rates. Elevated oxidative stress alters the maturation process of oocyte cytoplasm. This mechanism is essential for the translocation of organelles, regulation of maternal mRNA, and epigenetic programming, all of which influence the embryo’s developmental capacity long after fertilization [44].

Biomarkers in follicular fluid robustly substantiate this mechanistic approach. Elevated concentrations of MDA and 4-hydroxynonenal (4-HNE) indicate widespread lipid degradation within the follicular milieu [47]. Elevated concentrations of 8-hydroxy-2′-deoxyguanosine (8-OHdG) indicate that DNA is subjected to damage from oxidative stress. Research repeatedly shows that follicles with increased oxidative markers produce fewer mature oocytes, exhibit worse fertilisation rates, and result in embryos with decreased implantation potential and morphokinetic quality [48]. These biochemical alterations are associated with a diminished mitochondrial membrane potential in oocytes, irregularities in calcium oscillation patterns during fertilisation, complications in blastocyst growth, and reduced rates of euploidy. Oocytes from oxidatively stressed follicles display reduced mitochondrial clustering around the meiotic spindle, misalignment of the spindle apparatus, displacement of cortical granules, and abnormal spindle morphology, all of which correlate with suboptimal embryo development and unsuccessful fertilization [49].

Certain patient demographics are predisposed to reproductive issues due to oxidative stress. Women of advanced maternal age have increased mitochondrial DNA mutations in granulosa cells, reduced antioxidant gene expression, and accumulated oxidative damage. Women diagnosed with PCOS exhibit metabolic inflammation, elevated insulin levels, and increased androgen levels [50]. These circumstances enhance NOX4 activity and induce granulosa cells to produce excessive ROS. Granulosa cells from endometriotic ovaries have diminished Nrf2 activity and are predisposed to oxidative death. Individuals with endometriosis accumulate iron-derived reactive oxygen species in the peritoneal and follicular milieus [51]. Obesity causes oxidative stress and mitochondrial malfunction in granulosa cells, hindering steroidogenesis and reducing oocyte competence via persistent low-grade inflammation, dyslipidaemia, and lipotoxicity [52]. Heavy metals, particulate matter, and endocrine-disrupting substances exemplify environmental exposures that induce chronic redox stress. They accumulate in ovarian tissue, impairing the functionality of granulosa cells. Ovarian stimulation techniques generate temporary oxidative damage. In granulosa cells, elevated oestradiol levels enhance mitochondrial respiration, increase metabolic demand, and activate aromatase activity [53]. Although metabolic acceleration is essential for folliculogenesis, some follicles may experience oxidative overload due to insufficient antioxidant capability. This is particularly significant for women who may possess compromised antioxidant systems due to metabolic diseases or diminished ovarian reserve. However, by carefully selecting the kind of gonadotropin, adjusting dosages, and implementing co-interventions, controlled ovarian stimulation may be improved to reduce redox imbalance.

Translational research has identified several methods to enhance IVF results by augmenting the antioxidant capability of granulosa cells. Antioxidants that target mitochondria, such as coenzyme Q10, enhance the efficiency of the respiratory chain and prevent electron leakage [54]. Resveratrol enhances the transcription of antioxidant genes regulated by Nrf2, stimulates the formation of new mitochondria, and activates *SIRT1*. Melatonin, naturally present in human follicular fluid, stabilises mitochondrial membranes, eliminates free radicals, and enhances the expression of SOD2 and GPX4 in granulosa cells. Metformin reduces mitochondrial reactive oxygen species via enhancing cellular sensitivity to insulin and activating AMPK [55]. This reduces oxidative and inflammatory stress, particularly in those with PCOS. NAD^+^ precursors (NMN and NR) enhance DNA repair and mitochondrial robustness by refilling intracellular NAD^+^ levels and reinstating sirtuin function. Numerous observational and randomised studies demonstrate that these therapies promote the development of high quality blastocysts, elevate oocyte maturation rates, and boost clinical pregnancy and live birth rates [56].

Antioxidants need meticulous calibration prior to therapeutic use. Certain ROS are beneficial; for instance, ovulation, steroidogenesis, early embryo cleavage, and cumulus expansion all rely on regulated oxidative signals. Excessive or improperly timed antioxidant supplementation may obstruct these physiological redox signals, so paradoxically compromising luteal support or follicular competency [57]. Thus, the most promising translational strategy is focused, mechanism-informed antioxidant treatment rather than indiscriminate supplementation. Personalised antioxidant administration customised to a patient’s ovarian environment may soon be achievable, supported by precision medicine strategies based on follicular fluid redox indicators, granulosa cell transcriptome profiles, or mitochondrial functional evaluations.

## 4. Ferroptosis in Granulosa Cells: Iron Homeostasis, Lipid Peroxidation, and Regulated Cell-Death Pathways in Ovarian Aging

This section discusses ferroptosis as a distinctive and ovarian-relevant mechanism for the regulated demise of granulosa cells. It does this by integrating iron metabolism, lipid peroxidation, mitochondrial dysfunction, and antioxidant inadequacy. Ferroptosis has recently been identified in the ovary as a primary and mechanistically unique kind of controlled cell death. Ferroptosis is defined by iron-dependent lipid peroxidation, the severe destruction of membrane phospholipids rich in PUFAs, the impairment of mitochondrial redox buffering, and the subsequent structural collapse of intracellular membranes [57]. Activation of caspases and release of cytochrome c are essential for apoptosis, while the RIPK1/RIPK3-MLKL pathways are responsible for necroptosis. Ferroptosis in GCs is a highly sensitive, redox-regulated phenomenon that is exacerbated by chemotherapy, metabolic stress, environmental factors, and ovarian aging. It is not just an alternate route to death [58]. The molecular profile significantly interacts with mitochondrial dysfunction, compromised antioxidant defences, glutathione depletion, dysregulated iron metabolism, and altered lipid remodelling, hence hindering follicular survival and oocyte competence.

### 4.1. Iron Trafficking, Ferritinophagy, and the Buildup of the Labile Iron Pool in Granulosa Cells

Granulosa cells regulate iron homeostasis by a dynamic and meticulously regulated mechanism that equilibrates iron input, storage, utilisation, and export. This is essential for follicular growth and steroid production. Iron is essential for heme production, DNA synthesis, mitochondrial respiration, and the function of many cytochromes and metabolic enzymes [59]. When this equilibrium is disrupted, iron transitions from a vital cofactor to a significant contributor to oxidative damage and ferroptotic cell death. Granulosa cells are particularly susceptible to iron-induced damage because to their abundance of mitochondria, active steroidogenic pathways, and continuous exposure to reactive oxygen species generated during folliculogenesis and ovulation [60].

Ferritin nanocages, composed of FTH1 and light-chain (FTL) subunits, securely sequester the majority of intracellular iron under normal physiological conditions. FTH1’s ferroxidase activity inhibits Fenton reaction by converting Fe^2+^ into the less reactive and stable Fe^3+^ state, suitable for storage [61]. Granulosa cells primarily obtain iron through transferrin receptor 1 (TFRC), which binds transferrin-Fe^3+^ complexes at the plasma membrane, internalises them via clathrin-mediated endocytosis, and transports iron into endosomal compartments where it is reduced and released into the cytosol through DMT1 (divalent metal transporter 1) [62]. Typically, exogenous iron is rapidly sequestered in ferritin, heme-containing enzymes, or iron-sulfur clusters. This maintains a little quantity of catalytically active Fe^2+^, referred to as the labile iron pool (LIP).

This meticulously calibrated mechanism, however, starts to deteriorate with age, when our metabolism falters, during illness, or under oxidative stress. The enlargement of the LIP renders the intracellular environment more reactive, hence increasing the propensity of granulosa cells to experience lipid peroxidation and ferroptosis [63]. The primary factor contributing to the accumulation of redox-active Fe^2+^ is the activation of NCOA4-mediated ferritinophagy, a specialised form of autophagy that targets ferritin for lysosomal destruction. NCOA4 functions as a cargo receptor by associating with the heavy-chain component of ferritin and transporting ferritin complexes to autophagosomes [64]. Lysosomes decompose ferritin, releasing substantial amounts of Fe^2+^ into the cytosol. This significantly enlarges LIPs and accelerates the generation of iron-mediated ROS. Ferritinophagy enables cells to transport iron for metabolic functions under normal conditions. Excessive ferritinophagy in granulosa cells, which may occur during inflammation, ovarian aging, PCOS, or chemotherapy-induced stress, results in persistent iron accumulation and prepares the cell for ferroptosis [65].

Iron metabolism is markedly impaired in aging ovaries, as shown by elevated oxidative stress, decreased ferritin storage capacity, reduced ferroportin-mediated export, and increased expression of TFRC. These alterations result in granulosa cells being saturated with iron, however they are unable to eliminate excess iron or detoxify [66]. The activity of *SIRT1* and Nrf2 diminishes with age, exacerbating this disease. *SIRT1* typically inhibits ferritinophagy by regulating autophagic flux and maintaining mitochondrial integrity. Nrf2, conversely, promotes the transcription of FTH1 and ferroportin, hence enhancing iron sequestration and exportation [67]. Upon the loss of either regulatory axis, cells progress towards iron accumulation, ferritin depletion, and heightened susceptibility to ferroptosis. Excess iron from retrograde menstruation accumulates in ovarian tissue and follicular fluid in pathological ovarian diseases, such as endometriosis, leading to persistent oxidative stress. Granulosa cells from endometriotic ovaries have a ferroptosis-prone phenotype, marked by elevated iron staining, increased NCOA4 expression, and reduced antioxidant defences. Despite the apparent abundance of follicles, PCOS induces metabolic inflammation and hyperandrogenism, disrupting iron metabolism and increasing ferritinophagy. This impairs the functionality of granulosa cells [60]. Chemotherapy agents such as cyclophosphamide induce significant oxidative damage, degrade ferritin, and liberate iron. This results in a “ferroptotic storm” that rapidly annihilates the ovarian reserve and depletes granulosa cells.

In the expanded LIP, excessive Fe^2+^ participates in Fenton processes that convert hydrogen peroxide into hydroxyl radicals. These radicals may initiate robust lipid-peroxidation cascades. The oxidative burst requiring iron accelerates membrane degradation, mitochondrial collapse, and the initiation of ferroptotic death pathways [68]. This is due to the abundance of polyunsaturated phospholipids, such as arachidonic and adrenic acid, in granulosa cells. Increased LIP impairs mitochondria, exacerbating follicular dysfunction. It inhibits electron transport, elevates mitochondrial reactive oxygen species (mtROS), and decelerates steroidogenesis. Thus, a primary mechanism via which granulosa cells shift from physiological homeostasis to ferroptotic vulnerability is the disruption of iron transport and ferritinophagy. The accumulation of the labile iron pool initiates lipid peroxidation, surpasses antioxidant defences, and results in the irreversible death of granulosa cells [69]. It is not only a chemical consequence of aging or inflammation. Consequently, ferroptosis serves as the molecular connection among oxidative stress, mitochondrial dysfunction, iron accumulation, and accelerated follicular atresia. This suggests that iron metabolism may serve as an effective approach to address ovarian aging, infertility, and the preservation of fertility.

### 4.2. Lipid Peroxidation Machinery: ACSL4, LPCAT3, Lipoxygenases, and the Ferroptotic Collapse of Granulosa-Cell Membranes

The uncontrolled peroxidation of membrane phospholipids rich in PUFAs is a characteristic biochemical marker of ferroptosis in granulosa cells. Ferroptosis is characterised by a devastating, iron-dependent breakdown of lipid bilayers, leading to irreversible membrane damage, mitochondrial distortion, and structural failure of intracellular organelles, unlike apoptosis, which emphasises caspase activation and nuclear fragmentation [70]. Granulosa cells are especially susceptible to lipid peroxidation because to their high mitochondrial density, active steroidogenic processes, and dynamic membrane remodelling during folliculogenesis. A critical factor in ferroptotic sensitivity is the system that governs the incorporation of PUFAs into phospholipids, their enzymatic and non-enzymatic oxidation, and their detoxification processes [71]. The enzyme ACSL4 (acyl-CoA synthetase long-chain family member 4) is initiated during the onset of ferroptosis. It converts free arachidonic acid (AA) and adrenic acid (AdA) into their respective acyl-CoA derivatives. Upon activation, these omega-6 polyunsaturated fatty acids serve as optimal substrates for phospholipid esterification and peroxidation [72]. ACSL4 functions as a “lipid gatekeeper,” regulating the composition of the cellular lipidome and establishing the conditions for ferroptotic vulnerability. Granulosa cells from aged ovaries, metabolically inflamed PCOS follicles, and ovaries exposed to chemotherapy have elevated ACSL4 expression, indicating a shift to a PUFA-rich membrane milieu that is more prone to ferroptosis [72].

Upon activation of ACSL4, LPCAT3 (lysophosphatidylcholine acyltransferase 3) catalyses the esterification of AA-CoA and AdA-CoA into phosphatidylethanolamine (PE) and phosphatidylcholine (PC) species. This phase of lipid remodelling significantly elevates the quantity of PUFA-PEs, which serve as the primary substrates that undergo oxidation during ferroptosis [73]. LPCAT3 is significantly expressed in steroidogenic tissues such as the ovary. It accelerates vesicular trafficking, steroid hormone production, and membrane fluidity. Its overexpression generates a milieu of lipid molecules susceptible to oxidative damage and enhances PUFA enrichment. Increased LPCAT3 activity in granulosa cells is associated with accelerated follicular atresia and reduced oocyte developmental competence, especially when antioxidant systems are compromised [74]. Following the accumulation of PUFA phospholipids in membranes, both enzymatic and non-enzymatic mechanisms accelerate their oxidation. Lipoxygenases (LOXs), namely ALOX15 and ALOX12, are essential enzymatic oxidants. These iron-dependent dioxygenases synthesise phospholipid hydroperoxides (PE-OOH) by preferentially incorporating hydroperoxy groups into PUFA-Pes [75]. PE-OOH represents the first stages of ferroptosis. Granulosa cells have heightened responsiveness to oxidative stimuli owing to their production of lipoxygenases in response to inflammatory cytokines (TNF-α, IL-1β), LH-mediated ovulatory signalling, and ER-stress pathways. Research demonstrates that ALOX15 is significantly elevated in follicular settings exposed to toxicants such diesel particulate matter or bisphenol A, as well as during ovarian aging, therefore elevating the risk of ferroptosis [60].

POR (cytochrome P450 oxidoreductase) facilitates the creation of lipid peroxides by transferring electrons to cytochrome P450 enzymes, which generate reactive oxygen species during steroid metabolism, similar to the function of lipoxygenases. Granulosa cells exhibit steroidogenic activity, hence they are significantly dependent on POR, increasing their susceptibility to inadvertent initiation of ferroptosis [76]. Excessive reactive oxygen species generated during oestradiol synthesis might interact with iron-mediated Fenton chemistry to accelerate non-enzymatic lipid peroxidation, resulting in the accumulation of significant quantities of reactive aldehydes (4-HNE, MDA). These electrophilic aldehydes modify proteins, mitochondrial membranes, and cytoskeletal components, therefore preparing cells for ferroptotic collapse [77]. When lipid hydroperoxide levels exceed the capacity of GPX4 and mitochondrial antioxidant enzymes, the process may perpetuate autonomously. In the absence of enzymes to regulate them, peroxidised polyunsaturated fatty acids initiate chain reactions that rapidly oxidise adjacent phospholipids. The cristae dissolve, the outer mitochondrial membranes become permeable, and the mitochondrial membranes compress while increasing electron density. These morphological alterations significantly diminish ATP synthesis, disrupt pyruvate metabolism, inhibit the synthesis of progesterone and oestrogen, and ultimately lead to the degradation of granulosa cells that sustain the egg [78].

It is crucial to acknowledge that when GPX4 malfunctions, granulosa cells lack robust mechanisms to combat lipid peroxidation. Recent study indicates that FSP1 functions as an antioxidant outside of mitochondria, inhibiting lipid peroxidation in the plasma membrane via recycling ubiquinol [79]. However, it seems that it is insufficiently effective in human ovarian granulosa cells to combat elevated iron or polyunsaturated fatty acid levels. Granulosa cells depend significantly on antioxidant enzymes regulated by GPX4 and Nrf2. When these enzymes malfunction, ferroptosis occurs unimpeded. The ACSL4-LPCAT3-ALOX15 axis serves as the biochemical pathway for ferroptosis in granulosa cells [80]. It regulates the oxidation of lipids, the accumulation rate of peroxides, and the rate of cellular apoptosis. This process explains the considerable increase in ferroptotic vulnerability in the ovary attributable to variables such aging, PCOS, endometriosis, obesity, and chemotherapy, all linked to impaired lipid metabolism and chronic oxidative stress. Lipid peroxidation is recognised as a vital and actionable target for the maintenance of ovarian reserve, owing to the lipidomic remodelling caused by these circumstances, which leads to a membrane structure particularly vulnerable to ferroptotic breakdown.

### 4.3. Glutathione Depletion, GPX4 Inactivation, and the Ferroptotic Execution Program in Granulosa Cells

The disruption of the glutathione-GPX4 defence axis, the primary biochemical mechanism that determines whether oxidative stress remains a manageable metabolic issue or escalates into lethal, lipid-induced cell death, is a critical molecular event in the progression of granulosa cells towards ferroptosis [81]. Glutathione is the primary thiol-based antioxidant inside cells. It serves as the primary constituent for GPX4, which is the only enzyme capable of directly degrading phospholipid hydroperoxides in cellular and mitochondrial membranes. This axis serves as the last line of defence for membrane integrity and the capacity of granulosa cells to sustain oocytes. Granulosa cells continuously degrade lipids, synthesise steroids, and regulate fluctuating amounts of reactive oxygen species resulting from mitochondrial and endocrine functions [82].

Glutathione synthesis requires a consistent supply of cysteine. Granulosa cells primarily acquire this substrate via system Xc^−^, which is composed of the xCT (SLC7A11) and SLC3A2 subunits. Glutamate-cysteine ligase and glutathione synthase rapidly convert extracellular cystine into cysteine and incorporate it into glutathione [83]. Granulosa cells possess the ability to detoxify hydrogen peroxide, inhibit lipid peroxidation, and maintain mitochondrial membrane potential stability throughout the physiologically intensive phases of follicular development and hormone synthesis. This is due to this mechanism maintaining substantial glutathione reserves [84]. However, aging ovaries, inflammatory microenvironments such as those present in endometriosis, hyperinsulinemic and hyperandrogenic states characteristic of PCOS, and chemotoxic injury from agents like cyclophosphamide or doxorubicin all impede the body’s ability to absorb cystine and synthesise the enzymes required for glutathione production. Ferroptotic execution is enabled by the cell’s reduced ability to neutralise accumulating phospholipid hydroperoxides when glutathione levels decline.

The regulation of System Xc^−^ is intricately linked to processes triggered by oxidative, inflammatory, or genomic stress. p53 directly inhibits the transcription of SLC7A11. This often occurs in granulosa cells subjected to DNA damage as a result of aging or metabolic disorders [85]. This impedes the entry of cystine and accelerates the degradation of glutathione. Nrf2 typically enhances glutathione synthesis by stimulating the expression of antioxidant genes such as SLC7A11, GCLC, and GCLM. As humans age, Nrf2 levels decline, inhibiting a significant compensatory mechanism [86]. Metabolic dysfunction elevates cytosolic glutamate concentrations, impeding cystine efflux and diminishing the efficacy of the antiporter. Inflammatory mediators such as TNF-α and IL-1β further destabilise xCT. These regulatory issues have resulted in a gradual decline in intracellular levels of glutathione and cysteine. This complicates the ability of granulosa cells to inhibit lipid peroxidation [87].

Ferroptosis is primarily induced by the selenoenzyme GPX4, which degrades lipid hydroperoxides in phospholipids and inhibits radical-mediated chain reactions that may otherwise compromise the membrane integrity. GPX4 preserves the fluidity of the plasma membrane, the functionality of the endoplasmic reticulum, the architecture of the cristae, and the structural integrity of mitochondrial membranes [88]. Elevated GPX4 levels enable granulosa cells to withstand significant oxidative stress without undergoing ferroptosis. However, a decrease in GPX4 activity leads to an uncontrollable accumulation of phospholipid hydroperoxides, beyond the biochemical threshold that distinguishes irreversible ferroptotic death from reversible oxidative stress. Numerous procedures are required to deactivate GPX4 [89]. When glutathione depletes, GPX4 becomes nonfunctional since it requires glutathione for its activity. Iron-dependent hydroxyl radicals oxidise the active selenocysteine residue of GPX4, hence diminishing the enzyme’s catalytic efficacy. As Nrf2 levels decline with aging, GPX4 transcription diminishes. Persistent endoplasmic reticulum stress further diminishes GPX4 levels by reducing overall protein synthesis. Chemotherapy markedly diminishes the intracellular half-life of GPX4 by accelerating its degradation via ubiquitin-mediated mechanisms. The loss of GPX4 represents a biological point of no return in all instances [90].

Phospholipid hydroperoxides build at a rate above the residual antioxidant capability when GPX4 activity declines. As these peroxides traverse membranes, they significantly alter the membrane structure. The cristae dissolve, mitochondria reduce in size, and their membranes achieve a high electron density [91]. Electrophiles derived from lipids, such as 4-HNE and MDA, modify proteins, enzymes, and components of the cytoskeleton. They also diminish ATP levels in cells and disrupt calcium homeostasis. Ferroptosis transpires without the conventional apoptotic markers, such as nuclear condensation and caspase activation. Conversely, a covert but catastrophic lipid-induced implosion occurs inside the cell [92]. The glutathione-GPX4 axis is crucial for granulosa cells due to their membranes’ high content of polyunsaturated fatty acids, which is essential for follicular growth and endocrine signalling. Due to their reliance on vigorous mitochondrial metabolism, they are more susceptible to lipid damage induced by ROS. Granulosa cells exhibit heightened vulnerability when GPX4 is compromised, since alternate ferroptosis-inhibitory pathways, such as the FSP1-CoQ10 system that safeguards plasma membranes in non-ovarian tissues, are comparatively underexpressed in these cells [81]. Any situation that diminishes GSH availability or GPX4 functionality, such as aging, undergoing cytotoxic therapy, experiencing metabolic inflammation, or being in a hazardous environment, may accelerate the demise of granulosa cells and follicular atresia. Table 1 shows all of the primary molecular reasons why ferroptosis occurs in human granulosa cells. These consist of iron metabolism, lipid peroxidation, and the GPX4-GSH detoxification mechanism.

### 4.4. Mitochondrial Dysfunction as a Driver and Amplifier of Ferroptosis in Granulosa Cells

Mitochondria are the primary component of the ferroptotic cascade in granulosa cells. They constitute the origin of oxidative stress, the biochemical enhancer of lipid peroxidation, and the structural target of ferroptotic injury [93]. Ferroptosis internally alters mitochondrial function, whereas apoptosis produces controlled, caspase-dependent cell death. The generation of ROS escalates, redox buffering is compromised, lipids in the inner membrane suffer peroxidation, and the organelle experiences degenerative shrinkage due to oxidative damage rather than coordinated signalling. Granulosa cells need healthy mitochondria since their functionality relies on mitochondrial ATP production, cholesterol transport, steroidogenic enzyme activation, and communication with the oocyte. When mitochondria cease to function correctly, ferroptosis becomes not only feasible but very probable, jeopardising the life of the follicles [94].

The progressive decline in efficiency of the electron transport chain is the first alteration in mitochondria that predisposes granulosa cells to ferroptosis. Aging, metabolic inflammation, environmental contaminants, and oxidative spikes during ovulation all impair complexes I and III. This results in electron leakage and excessive production of superoxide [95]. Mitochondrial antioxidant enzymes typically convert superoxide into hydrogen peroxide. Peroxiredoxins and glutathione peroxidases further decompose hydrogen peroxide. This system becomes overburdened when glutathione levels decrease or GPX4 activity diminishes. Hydrogen peroxide accumulates in the mitochondrial matrix and interacts with Fe^2+^ to generate hydroxyl radicals that damage the polyunsaturated phospholipids in mitochondrial membranes. These first oxidative processes initiate lipid peroxidation far in advance of any observable morphological alterations [96].

Granulosa cells struggle to compensate for issues with mitochondrial respiration, since their steroidogenic activity relies on the efficient functioning of StAR and CYP11A1 enzymes to transport cholesterol into the mitochondria. Impaired mitochondrial respiration results in reduced energy availability and complications in steroid production [94]. The cell’s attempt to replenish its antioxidant reserves results in a metabolic failure that elevates NADPH levels in the cytosol, hence exacerbating the depletion of redox buffering essential for halting ferroptotic development. Mitochondrial iron metabolism significantly elevates the incidence of ferroptosis [97]. Mitochondria sequester redox-active metal ions due to their abundance of iron-sulfur clusters, heme groups, and iron-dependent enzymes. Iron is released from compromised heme proteins, iron-sulfur clusters degrade under oxidative or inflammatory stress, and mitochondrial ferritin is unable to retain the released Fe^2+^. The accumulation of labile iron inside the mitochondria accelerates Fenton chemistry, generating hydroxyl radicals in proximity to PUFA-rich inner-membrane phospholipids. Oxidative phosphorylation is impaired, the architecture of the cristae is disrupted, and a localised, self-propagating wave of lipid peroxidation disseminates into the cytoplasm [98].

As lipid peroxides accumulate inside mitochondria, profound ultrastructural alterations indicative of ferroptosis occur. Mitochondria become diminutive and hypercondensed when their inner membranes are thick and irregular, resulting in the loss of cristae [99]. These alterations illustrate the distinctions between ferroptosis and necrosis, the latter of which induces swelling, and early apoptosis, which is linked to cytochrome-c and cellular remodelling. The breakdown of cristae reduces mitochondrial membrane potential and impairs ATP generation, creating a metabolic environment unsuitable for granulosa-cell maintenance of the oocyte. As calcium homeostasis diminishes, oxidative phosphorylation becomes more erratic, and the mitochondrial permeability transition occurs independently of apoptosis or caspase involvement. This induces the release of ROS into the cytosol and accelerates lipid-peroxidation cycles [78].

Mitochondrial malfunction diminishes the efficacy of mitophagy. This is a crucial quality control mechanism that often eliminates defective mitochondria before they disrupt cellular equilibrium. The PINK1-Parkin pathway, responsible for identifying depolarised mitochondria for autophagic degradation, has diminished activity in aged granulosa cells due to reduced activity of PGC-1α, *SIRT1*, and AMPK. This leads to the accumulation of defective mitochondria, each generating a milieu of oxidative and iron-mediated damage that perpetuates the ferroptotic cycle [100]. The dysfunction of mitochondrial turnover is one of the first molecular issues identified in aged ovarian tissue. This is considered indicative of the transition from compensated oxidative stress to active ferroptotic susceptibility. The metabolic requirements of granulosa cells render them more susceptible. These cells provide the oocyte with pyruvate, lactate, amino acids, and antioxidants via gap junctions and transzonal projections [101]. When mitochondria cease functioning, the oocyte loses communication with granulosa cells, becoming it more susceptible to ROS, depleting its ATP precursors, and depriving it of glutathione. Ferroptosis mostly begins in the granulosa cell compartment, indirectly provoking metabolic stress and meiotic instability in the oocyte. Mitochondrial failure is a significant contributor to ferroptosis in granulosa cells, rather than a trivial factor [102]. It impedes detoxification pathways by diminishing cellular antioxidants, resulting in excessive ROS formation, heightened lipid peroxidation due to iron release from mitochondria, and the cessation of both energy metabolism and steroidogenesis. Ferroptosis is an inevitable consequence of the degradation of mitochondrial structure and redox capability. Granulosa cells depend significantly on mitochondria for their endocrine, metabolic, and defensive roles. Ferroptosis adversely affects follicular survival, and maintaining mitochondrial health seems to be an effective strategy to combat infertility, chemotherapy-induced damage, and ovarian aging [60].

### 4.5. Experimental and Clinical Evidence of Ferroptosis in Ovarian Models of Aging, PCOS, Chemotherapy, and Premature Ovarian Insufficiency

Ferroptosis is a principal mechanism behind granulosa cell depletion in several ovarian diseases, rather than a secondary or incidental occurrence, as corroborated by a growing corpus of data from human clinical samples, in vitro granulosa cell investigations, animal models, and high-resolution omics technologies [60,99]. Ferroptosis consistently constitutes the earliest, most significant, and most detrimental pathway leading to follicular atresia and the reduction of ovarian reserve, regardless of the underlying cause whether it be aging, metabolic dysfunction, environmental toxins, inflammatory stress, or chemotherapeutic agents. This section discusses the significance of ferroptosis in medicine and provides a concise summary of findings indicating it is a physiologically conserved response in the ovary [60,103].

Human IVF granulosa cells, offering the most direct view into ferroptosis in the human ovary, give significant data. These alterations are associated with diminished levels of the GPX4 protein, reduced glutathione, and the accumulation of lipid-peroxidation byproducts such as 4-HNE and MDA in follicular fluid. In old IVF granulosa cells, mitochondrial ultrastructure often displays shrunken, hyper-condensed mitochondria devoid of cristae characteristic indicators of ferroptosis [104]. Ferroptosis is a therapeutically relevant predictor of reproductive success, shown by the association between these molecular markers and inadequate oocyte maturation, decreased cytoplasmic competence, impaired blastocyst development, and lower euploidy rates.

Granulosa cells from women with PCOS demonstrate significant overexpression of TFRC and ACSL4, increased lipid ROS buildup, and reduced GPX4 expression compared to controls. Hyperandrogenism and hyperinsulinemia are the primary etiological factors of PCOS [105]. They exacerbate mitochondrial dysfunction and induce the generation of reactive oxygen species from NOX4. These metabolic anomalies provide an intracellular environment favourable to ferroptosis by suppressing Nrf2 activity and reducing glutathione production, along with ongoing inflammatory signalling [106]. PCOS follicles often produce dysmature or developmentally incompetent oocytes, although looking abundant on ultrasound. This contradiction is explained by ferroptotic damage inside the granulosa-cell compartment rather than the amount of follicles. Ferroptosis has been shown as an early and progressive characteristic in animal models of ovarian aging [60]. Aged mice have a unique ferroptotic signature in granulosa cells, marked by the downregulation of Gpx4, *SIRT1*, and Nrf2, and the overexpression of Acsl4, Tfrc, and Ncoa4. Prior to the observable histologic depletion of follicles, lipid peroxidation products accumulate, indicating that ferroptosis functions as both a molecular precursor and a result of follicular atresia. In vivo genetic or pharmacological suppression of ferroptosis safeguards ovarian reserve, enhances oocyte quality, and extends reproductive longevity, indicating both correlation and causation [103].

Models of ovarian damage induced by chemotherapy, particularly those using cyclophosphamide, cisplatin, and doxorubicin, may provide the most compelling evidence that ferroptosis is the primary mechanism behind granulosa cell depletion. The substantial oxidative and genotoxic stress induced by these drugs leads to the deterioration of the mitochondrial antioxidant network, suppression of GPX4, and rapid depletion of glutathione [107]. Chemotherapy induces significant ferritinophagy, resulting in enlarged lipid droplets and exceeding the detoxifying capacity of lipid peroxides. Granulosa cells exposed to these chemicals exhibit the distinctive morphological features of ferroptosis, including mitochondrial shrinkage, condensation, and cristae loss. Ferroptosis inhibitors, including ferrostatin-1, liproxstatin-1, iron chelators, and GPX4-stabilizing agents, provide significant protection to ovarian follicles in these animals, but apoptosis inhibitors do not. The findings suggest that the primary mechanism via which chemotherapy causes early ovarian insufficiency is ferroptosis, not apoptosis [60,108].

Toxicological and environmental models augment this framework. Granulosa cells exposed to endocrine-disrupting substances, including bisphenol A, phthalates, diesel particulate matter, and heavy metals, exhibit indications of ferroptosis, characterised by elevated lipid ROS levels, decreased GPX4 levels, and activation of lipoxygenase pathways. Follicles exposed to these toxicants exhibit indications of ferroptotic degeneration, including increased atresia and diminished oocyte competence [109]. Ferroptosis is a ubiquitous biochemical reaction to many environmental stressors, as shown by the amplification of these effects triggered by ambient iron or contaminants that might produce ROS. Ferroptosis is a conserved biological failure mechanism of granulosa cells, and is characterised by a consistent ferroptotic signature iron overload, GSH depletion, GPX4 suppression, ACSL4/LPCAT3 activation, and mitochondrial collapse observed across diverse situations. Ferroptosis is a crucial biological route in reproductive medicine, characterised by its early beginning, cross-species repeatability, and strong association with oocyte quality. Understanding the processes of follicular atresia not only elucidates this process but also provides a basis for targeted treatment strategies to preserve fertility and mitigate ovarian aging.

## 5. *SIRT1* in Granulosa Cell Homeostasis

### 5.1. SIRT1 as a Central Regulator of Oxidative Stress and Granulosa-Cell Identity

This section discusses *SIRT1*, the primary metabolic and redox-sensitive regulator of granulosa cell viability, mitochondrial quality control, and resistance to oxidative stress-induced ferroptosis. *SIRT1* is a distinctive and crucial component of granulosa cell physiology, serving as a molecular regulator that equilibrates metabolic status, oxidative stress response, mitochondrial function, and reproductive capability [110]. Granulosa cells inhabit a highly dynamic milieu during follicular development, ovulation, and luteinisation, and are characterised by fluctuating levels of ROS, energy requirements, endocrine signals, and paracrine communications. *SIRT1* allows granulosa cells to comprehend these fluctuations and convert them into transcriptional programs that are either protective or adaptive [111]. A NAD^+^-dependent deacetylase’s activity directly correlates with the cell’s metabolic condition. Elevated NAD^+^ levels enhance *SIRT1* functionality, facilitating antioxidant responses and maintaining mitochondrial health. Low NAD^+^ levels, which occur with aging, inflammation, metabolic load, or chemotoxic damage, diminish the efficacy of *SIRT1*, rendering granulosa cells more susceptible to redox imbalance and ultimately to ferroptotic vulnerability [112]. The genomic activities of transcription factors regulated by *SIRT1* dictate the identity and potency of granulosa cells. *SIRT1* establishes the first defence against ROS accumulation and maintains mitochondrial membrane potential by enhancing the transcription of antioxidant enzymes such as SOD2 and catalase via the deacetylation of FOXO3 [113]. The *SIRT1*-dependent activation of FOXO3 is crucial for genomic integrity during follicular development, since FOXO3 regulates genes associated with DNA repair, apoptosis prevention, and oxidative stress management. *SIRT1* activation of PGC-1α enhances the expression of genes associated with mitochondrial biogenesis, resulting in the generation of new, functioning mitochondria capable of efficient oxidative phosphorylation [114]. This contact facilitates granulosa cells in generating sufficient ATP for steroidogenesis, maintains the connection to the oocyte via gap junctions, and alters the cytoskeletal structure during follicular enlargement. Table 2 elucidates *SIRT1*’s primary molecular functions in granulosa cells, including its regulatory, metabolic, and anti-ferroptotic activities.

*SIRT1* serves as a potent anti-inflammatory agent by deacetylating NF-κB, therefore inhibiting the production of pro-inflammatory cytokines that may otherwise exacerbate oxidative damage and disrupt steroidogenic pathways [115]. *SIRT1* regulates inflammation to maintain a milieu conducive to the differentiation of granulosa cells and the appropriate functioning of endocrine activities. Moreover, inhibiting p53 prevents the premature activation of stress-response pathways that regulate cystine entry via SLC7A11 [116]. This inhibits the cell’s synthesis of glutathione and safeguards it from lipid peroxidation, an indicator of ferroptosis. The p53–*SIRT1* axis demonstrates that stresses increasing p53 levels, such as aging and chemotherapy, may swiftly reduce *SIRT1* activity and make granulosa cells susceptible to ferroptotic death [117]. It directly links genomic monitoring to vulnerability to ferroptosis. Granulosa cells, being the primary metabolic interface of the oocyte, must transport pyruvate, lactate, amino acids, nucleotides, and antioxidant compounds across gap junctions and transzonal projections. These metabolic processes need mitochondria to function in a highly regulated manner [118]. *SIRT1* ensures that mitochondria maintain optimal membrane potential, appropriate cristae architecture, enough respiratory chain efficiency, and a balanced fusion-fission dynamics. *SIRT1* facilitates mitophagy by activating PINK1 and Parkin, therefore eliminating damaged mitochondria that would otherwise produce excessive ROS and release iron and oxidised lipids. In the absence of this quality-control mechanism, granulosa cells become susceptible to ferroptosis due to elevated lipid-peroxide levels in certain regions, mitochondrial dysfunction, and the accumulation of oxidative damage [119].

*SIRT1* mitigates these hazards by ensuring the proper importation of mitochondrial cholesterol and maintaining chlorogenic flow via steroidogenic enzymes such as CYP11A1 and CYP19A1. A decline in *SIRT1* levels results in increased ROS generation, granulosa cells lose their capacity to grow concurrently with the oocyte, steroidogenesis becomes less reliable, and mitochondrial membranes are more susceptible to oxidative stress-induced damage [24]. These disturbances impair the embryo’s developmental capacity, the configuration of the meiotic spindle, and the maturation of the oocyte’s cytoplasm. The dependence of granulosa cells on *SIRT1* is particularly pronounced during reproductive aging [120]. With advancing age, our NAD^+^ levels decline, resulting in diminished *SIRT1* activity. This diminishes the efficacy of mitophagy, undermines our antioxidant defences, and leads to the accumulation of dysfunctional mitochondria. Before follicular depletion, this mitochondrial degradation acts as an early molecular marker of ovarian aging. Prior to the histological manifestation of overt follicular atresia, aged granulosa cells have transcriptional profiles suggestive of impaired FOXO3 activity, reduced SOD2 levels, decreased PGC-1α signalling, and sustained activation of stress pathways. This constellation identifies ferroptosis as the primary mechanism of cell death.

### 5.2. SIRT1, Mitochondrial Fitness, and the Prevention of ROS-Driven Follicular Decline

During folliculogenesis, granulosa cells undergo substantial lipid remodelling and steroidogenic activation, which naturally increases the generation of ROS. *SIRT1* alleviates these hazards by facilitating the appropriate importation of mitochondrial cholesterol and sustaining chlorogenic flow via steroidogenic enzymes like CYP11A1 and CYP19A1 [121]. A reduction in *SIRT1* levels leads to an increase in ROS production, causing granulosa cells to lose their ability to develop alongside the oocyte, a decrease in the reliability of steroidogenesis, and an increased vulnerability of mitochondrial membranes to oxidative stress-induced damage [60,122]. These disruptions hinder the embryo’s growth potential, the arrangement of the meiotic spindle, and the maturation of the oocyte’s cytoplasm. The reliance of granulosa cells on *SIRT1* is especially significant during reproductive aging. As we age, our NAD^+^ levels decrease, leading to reduced *SIRT1* activity. This reduces mitophagy effectiveness, compromises our antioxidant defences, and results in the buildup of defective mitochondria [123]. This mitochondrial degradation serves as an early molecular indicator of ovarian aging prior to follicular depletion. Before the histological evidence of pronounced follicular atresia, aged granulosa cells have transcriptional patterns that suggest decreased FOXO3 activity, lower SOD2 levels, impaired PGC-1α signalling, and persistent activation of stress pathways. This constellation designates ferroptosis as the principal mechanism of cellular demise.

### 5.3. SIRT1 Downregulation in Ovarian Aging and Metabolic Disease

The decline of *SIRT1* is one of the first and most important molecular events in the aging of the ovaries. It happens before the obvious loss of follicles and changes the biochemical environment in which granulosa cells slowly lose their ability to handle oxidative stress, keep mitochondria healthy, and resist ferroptosis [24]. As ovaries get older, they lose intracellular NAD^+^, which is a metabolite that *SIRT1* needs to work. This stops this important deacetylase from working. This drop in NAD^+^ levels is caused by more inflammatory signalling, less efficient mitochondria, DNA damage that triggers PARP1, and less nicotinamide phosphoribosyltransferase (NAMPT), which is the enzyme that slows down the process of salvaging NAD^+^. When NAD^+^ levels drop, granulosa cells can’t activate FOXO3, PGC-1α, and other important mitophagy and antioxidant pathways. *SIRT1* also becomes less active. This causes mitochondrial quality control to slowly break down [124].

FOXO3 becomes hyperacetylated in aging granulosa cells because *SIRT1* is not working. This makes it less able to control the transcription of SOD2, catalase, and thioredoxin-dependent detoxifying enzymes. This changes the redox balance in a way that makes ROS build up more, especially in mitochondria where electron-transport chain problems are already making oxidative stress worse [125]. Granulosa cells have fewer working mitochondria because they can’t activate PGC-1α. This also slows down TFAM-driven mtDNA replication, makes it harder to put together the respiratory chain, and makes it harder for mitochondria to grow. This drop shows that the production of ROS has reached a point where it is greater than the body’s ability to get rid of it. This starts lipid-peroxidation events that slowly get granulosa cells ready for ferroptosis [126]. Another important effect of *SIRT1* downregulation as we get older is that it messes up mitochondrial proteostasis. Damaged mitochondria build up instead of being replaced when *SIRT1* doesn’t activate FOXO3 and PINK1-Parkin mitophagy. These mitochondria that aren’t working right leak Fe^2+^ from unstable iron-sulfur clusters, make dangerous aldehydes, and let out too much superoxide. This buildup of iron inside mitochondria drives the Fenton reaction, which makes hydroxyl radicals that directly oxidise phospholipids in the inner membrane [127]. Over time, this leads to widespread mitochondrial shrinkage, loss of cristae, collapse of the membrane potential, and the formation of electron-dense mitochondrial morphologies that are typical of ferroptosis. As the ovaries age, ferroptotic signatures like GPX4 suppression, increased ACSL4 activity, and the expansion of the labile iron pool within follicular cells become more common. This is because older granulosa cells can’t get rid of these mitochondria [128].

*SIRT1* decline also has a big effect on the endocrine function of granulosa cells. Aromatase (CYP19A1), which is needed to make oestradiol, is controlled by *SIRT1*-dependent signalling through the PGC-1α and estrogen-receptor coactivator pathways [129]. When *SIRT1* activity is lower in older granulosa cells, it messes up these regulatory networks, making it harder to make oestradiol even when FSH stimulation is normal. Lower levels of oestradiol make oocytes less mature, make follicular structures less stable, and make granulosa cells less likely to survive. So, the endocrine problems caused by a drop in *SIRT1* levels are directly linked to the lower quality of oocytes with age, the higher rates of meiotic spindle abnormalities, and the higher rates of aneuploidy in human embryos [130]. Metabolic disorders speed up the process of *SIRT1* downregulation and make the ovaries age faster than they normally would. Low-grade inflammation, high free fatty acids, and chronic hyperinsulinemia all work together to stop *SIRT1* transcription and its deacetylase activity in PCOS. This makes the mitochondria not work right, raises ROS levels, makes lipid peroxidation worse, and stops granulosa cells from differentiating [131]. This helps to explain why women with PCOS often make a lot of follicles but not many high-quality oocytes. Lipotoxicity, especially from palmitate and linoleate, raises intracellular ceramides and ER stress in PCOS granulosa cells. This lowers *SIRT1* activity and speeds up ferroptotic priming. Follicular fluid in endometriosis has high levels of iron and inflammatory cytokines, which cause oxidative stress that is too strong for lower *SIRT1* levels to make up for. This leads to a long-term redox imbalance and problems with folliculogenesis [132].

This problem gets worse when you are outside. Endocrine-disrupting chemicals like bisphenol A, phthalates, and particulate pollutants lower *SIRT1* by changing its transcription and making its chromatin less accessible. They do this through epigenetic changes like promoter methylation and histone acetylation. These changes make granulosa cells more likely to undergo ferroptosis, lower the quality control of mitochondria, stop the production of glutathione, and promote the dysregulation of iron. The fact that age-related, metabolic, and environmental stressors all affect the *SIRT1* pathway shows how important this regulatory axis is for the health of granulosa cells and how easily it can be broken. *SIRT1* downregulation causes the ovarian microenvironment to have more ROS, weaker antioxidant barriers, dysfunctional mitochondria, disrupted steroidogenesis, and uncontrolled lipid peroxidation. This environment not only speeds up follicular atresia, but it also makes oocytes less able to develop, which lowers their chances of fertilisation, slows down embryonic development, and lowers the rates of implantation and live birth in in vitro fertilisation.

### 5.4. SIRT1 as a Barrier Against Chemotherapy-Induced Oxidative Collapse and Ferroptosis

Chemotherapy is one of the most damaging things that can happen to granulosa cells. It overwhelms their antioxidant defences and kills them quickly, which can lead to early ovarian insufficiency. *SIRT1* is the main molecular shield that decides if chemotoxic stress can be absorbed, fixed, or becomes permanent [133]. This is true for all of the defence systems that granulosa cells have. Its loss during chemotherapy exposure signifies a molecular inflection point, subsequent to which unregulated ferroptotic cascades, lipid peroxidation, and mitochondrial dysfunction transpire. Understanding the behaviour of *SIRT1* in chemotoxic environments elucidates the heightened susceptibility of granulosa cells to chemotherapy and the emerging efficacy of enhancing *SIRT1* activity as a protective strategy for the ovaries [134].

Chemotherapeutic drugs such as cyclophosphamide, cisplatin, and doxorubicin induce a significant increase in DNA damage, mitochondrial reactive oxygen species, lipid peroxidation products, and protein adducts within minutes to hours of exposure. To fix DNA damage, these drugs turn on PARP1, which quickly uses up NAD^+^ [135]. *SIRT1*’s deacetylase activity only works when NAD^+^ is present. When NAD^+^ pools are quickly used up, *SIRT1* stops working. Granulosa cells lose their main antioxidant and mitochondrial-support programs when they are most likely to be hurt by oxidative stress because they can’t deacetylate FOXO3 and PGC-1α [136]. ROS accumulates quickly, especially in the mitochondrial matrix, where chemotherapy damages the electron transport chain, causing a lot of electrons to leak out and making superoxide and hydrogen peroxide. This ROS quickly goes beyond the body’s ability to detoxify itself if *SIRT1* doesn’t activate antioxidant enzymes [137]. In addition, turning off *SIRT1* messes with quality control and the turnover of mitochondria. Normally, PGC-1α activity and *SIRT1*-dependent FOXO3 signalling keep mitophagy going through PINK1 and Parkin. This lets granulosa cells get rid of mitochondria that have been damaged by chemicals. But mitophagy stops when *SIRT1* stops working. The buildup of damaged mitochondria creates a microenvironment where oxidative and iron-mediated damage can happen. These mitochondria boost the Fenton reactions inside mitochondria that make hydroxyl radicals by letting Fe^2+^ out of unstable iron-sulfur clusters [138]. The result is rapid, uncontrolled lipid peroxidation that affects phospholipids in the outer membrane, cardiolipins in the inner membrane, and cytosolic membranes nearby. After just a few hours of chemotherapy, the signs of ferroptosis, such as mitochondrial shrinkage, cristae collapse, and hyper-dense membranes, become clear.

The inhibition of GPX4, the principal enzyme that inhibits ferroptosis, constitutes a notably critical molecular consequence of *SIRT1* inactivation during chemotherapy. Both transcriptional and post-translational mechanisms lower GPX4 levels during chemotoxic stress. *SIRT1* usually keeps the pathway for making glutathione going by bringing in cystine and directly supporting GPX4 by stabilising Nrf2 [139]. When *SIRT1* isn’t working, glutathione pools break down, cystine uptake through SLC7A11 goes down because p53 is always active, and Nrf2 can’t move to the nucleus. Granulosa cells cannot stop the buildup of lipid hydroperoxides because GPX4 is both blocked and broken down quickly. Even if there are apoptosis inhibitors, ferroptosis can’t be stopped once GPX4 loss reaches a certain level [140]. *SIRT1* also controls how cells respond to stress in the nucleus, which changes a lot during chemotherapy. When *SIRT1* is active, it stops the production of cytokines, stops NF-κB-mediated inflammation, and stops the upregulation of oxidative mediators that make granulosa-cell membranes unstable. When *SIRT1* is turned off, NF-κB becomes hyperactivated, which leads to more TNF-α, nitric oxide, and inflammatory ROS being made. These inflammatory signals slow down the work of steroidogenic enzymes and speed up lipid peroxidation. So, the drop in *SIRT1* caused by chemotherapy starts an external inflammatory cascade that makes cells more likely to undergo ferroptosis, as well as an internal mitochondrial stress response [141].

Experimental data consistently validate this molecular model. Chemotherapy causes ovarian tissue to lose *SIRT1* protein levels quickly, followed by rises in lipid ROS, iron buildup, mitochondrial fragmentation, and GPX4 depletion. When *SIRT1* is activated by drugs like melatonin, resveratrol, NAD precursors (NMN, NR), or *SIRT1*-specific agonists, these bad things happen a lot less often. When *SIRT1* is activated, it keeps glutathione levels stable, stabilises GPX4, boosts antioxidant responses through FOXO3 and PGC-1α, and restores mitochondrial biogenesis [142]. These protective effects partially inhibit premature ovarian insufficiency, preserve follicular architecture, and reduce granulosa cell apoptosis. The significance of *SIRT1* in ferroptotic defence is underscored by the inability of ferroptosis inhibitors to fully reverse chemotherapy-induced ovarian damage without at least a partial restoration of *SIRT1* function. Consequently, the biochemical threshold that differentiates irreversible ferroptotic ovarian collapse from reversible chemotherapy-induced stress is *SIRT1*. In addition to showing that targeting the *SIRT1*-NAD^+^ axis could be a good way to protect ovarian reserve in cancer patients, the fact that it quickly goes down during chemotoxic exposure explains why granulosa cells are so sensitive to chemotherapy. *SIRT1*’s role in preventing chemotherapy-induced ferroptosis is one of the best examples of how metabolic and epigenetic regulators affect ovarian longevity in the larger context of reproductive medicine.

### 5.5. Pharmacologic and Nutritional Activation of SIRT1: Therapeutic Opportunities for Ovarian Protection

The ability to boost *SIRT1* activity with drugs opens up a big therapeutic window in reproductive medicine. This window could help stop oxidative damage, ferroptosis, agiing, and stress on ovarian function. *SIRT1* activation offers a unique and complete way to restore granulosa-cell homeostasis in many pathological conditions because it includes NAD^+^ availability, mitochondrial biogenesis, redox buffering, ferroptosis suppression, and steroidogenic regulation [117,141]. Increasing *SIRT1* activity brings back the antioxidant, anti-apoptotic, and anti-ferroptotic programs that granulosa cells need to stay alive. This is especially important because oxidative stress, metabolic demand, and mitochondrial vulnerability are all very sensitive in these cells. Resveratrol is one of the best-known *SIRT1* activators. It is a polyphenolic compound that boosts *SIRT1* levels directly and indirectly by activating AMPK, improving NAD^+^ recycling, and changing how mitochondria work. Resveratrol increases the activity of SOD2 and catalase, stimulates the expression of antioxidant genes, and restores mitochondrial biogenesis at the molecular level by encouraging the deacetylation of FOXO3 and PGC-1α [143]. Because of this, there is less ROS leakage and more healthy mitochondria that work well in the electron transport chain. Resveratrol stops mitochondria from depolarising, keeps the cristae structure, and lowers the amount of lipid ROS that builds up in granulosa cells when they are under oxidative stress. These effects are consistent with stopping the start of ferroptosis at the mechanistic level. Resveratrol protects glutathione levels and stops GPX4 from breaking down, which strengthens the glutathione-GPX4 axis. This axis is the main biochemical barrier against ferroptosis.

Melatonin is one of the strongest *SIRT1*-boosting substances found in ovarian cells. It is especially high in follicular fluid compared to the rest of the body. Melatonin makes FOXO3, PGC-1α, and NF-κB deacetylation stronger by boosting *SIRT1* transcription, stabilising the protein, and moving it to the nucleus. This leads to a better mitochondrial membrane potential, higher mitophagy efficiency, and a big drop in mitochondrial ROS output [144]. Melatonin keeps iron levels in the mitochondria stable during granulosa-cell ferroptosis by stabilising iron-sulfur cluster proteins and lowering ferritinophagy. This stops the initiation of the iron-dependent lipid-peroxidation cascade. Melatonin also protects GPX4 from oxidative inactivation by increasing the production of glutathione through *SIRT1*-dependent pathways that raise the levels of SLC7A11 and GCLC [145]. All of these effects make melatonin a very strong physiological modulator of *SIRT1*, which has direct clinical implications for endometriosis, PCOS, aging ovaries, and ovarian decline caused by chemotherapy. Metformin is another important drug that can activate *SIRT1*. It mostly works through the AMPK-NAD^+^ axis. Metformin activates AMPK, which boosts *SIRT1*’s enzymatic activity and speeds up the regeneration of NAD^+^ [146]. This makes the mitochondrial respiratory chain work better, stops the buildup of lipotoxic species, and makes β-oxidation in granulosa cells work better. These changes in metabolism stop peroxidative damage to mitochondrial membranes and lower the amount of ROS that is made. Metformin-driven *SIRT1* activation restores mitochondrial function, lowers intracellular ROS, enhances steroidogenic gene expression, and stabilises GPX4 in PCOS granulosa cells, where lipid toxicity, hyperinsulinemia, and inflammatory stress converge. This molecular constellation makes ferroptotic susceptibility much lower.

One of the best and most direct ways to boost *SIRT1* activity may be to bring NAD^+^ levels back up inside cells, especially with precursors like nicotinamide riboside and nicotinamide mononucleotide. Even in old or damaged granulosa cells, adding NAD^+^ back to them reactivates *SIRT1*, which brings FOXO3 activity back to life, encourages the growth of new mitochondria, speeds up mitophagy, and stops the p53-mediated repression of SLC7A11 [136]. These changes keep GPX4 activity up, stop lipid-peroxide buildup, and keep glutathione production going. Animal studies have shown that NR and NMN can save old ovaries by improving the quality of oocytes, restoring the metabolic cooperation between granulosa cells and oocytes, and lowering the early ferroptotic signatures that are a sign of ovarian aging. Quercetin, berberine, spermidine, and synthetic *SIRT1* activators like SRT2104 are other small molecules that have been shown to improve mitochondrial quality control, boost *SIRT1* signalling, lower inflammation, and lower oxidative stress in granulosa and ovarian somatic cells. These substances all work in the same way to control the FOXO3-PGC-1α axis, mitochondrial turnover, and NAD^+^ availability. This makes *SIRT1* an even better target for drugs that can help keep ovarian reserve and fertility.

## 6. Nrf2 as Master Regulator of Antioxidant Defense

### 6.1. The Nrf2-Keap1 Redox-Sensing System as the Central Antioxidant Switch in Granulosa Cells

The Nrf2-Keap1 axis is the main redox-sensing structure in granulosa cells. It is the molecular interface that detects changes in oxidative stress and coordinates a genome-wide transcriptional response that protects the cell from ROS buildup, lipid peroxidation, electrophilic injury, and metabolic instability [147]. Granulosa cells in the ovarian follicle are always under a lot of oxidative stress because of mitochondrial respiration, steroidogenic processes, inflammatory mediators, and cumulus-oocyte interactions. This makes the Nrf2-Keap1 system very important. It is the main switch that decides whether granulosa cells can keep their redox balance or go into oxidative dysfunction, which shortens the life of the follicle [50,60].

Keap1 (Kelch-like ECH-associated protein 1) tightly stops Nrf2 from working in healthy conditions by acting as a substrate adapter for a Cul3-dependent E3 ubiquitin ligase complex. This interaction keeps newly made Nrf2 levels low by promoting ongoing ubiquitination and proteasomal degradation [148]. A group of very reactive cysteine residues (especially Cys151, Cys273, and Cys288) act as redox-sensitive switches to make Keap1’s regulatory role possible. These cysteines are very sensitive to ROS and electrophilic species, and when they are changed, Nrf2 is released. These cysteine sensors work as early warning systems for oxidative imbalance in granulosa cells, where steroidogenic enzymes make huge amounts of hydrogen peroxide and mitochondrial complexes I and III leak superoxide all the time [147]. When oxidative or electrophilic stress is higher than normal, reactive cysteine residues in Keap1 may be oxidised, S-alkylated, S-nitrosylated, or form disulphide bonds. These changes make Keap1’s structure worse, which makes it less likely to bind to the Nrf2 Neh2 domain. Because of this, Nrf2 doesn’t get ubiquitinated and builds up in the cytoplasm. When Keap1 stops keeping Nrf2 down, it moves into the nucleus. This is made easier by the fact that NLS in the Nrf2 structure become visible. This quick change from cytosol to nucleus is the most important thing that happens to activate the antioxidant defence program in granulosa cells [149].

Nrf2 forms heterodimers with small Maf proteins in the nucleus and binds to AREs in the promoter regions of many genes that are involved in redox buffering, detoxification pathways, glutathione synthesis, thioredoxin recycling, heme metabolism, and iron storage. This transcriptional network is huge [150]. Nrf2 activates genes that neutralise hydrogen peroxide (like catalase and peroxiredoxins), detoxify lipid hydroperoxides and quinones (like NQO1 and GPX family members), replenish glutathione pools (GCLC, GCLM, GSS), recycle NADPH, and help proteins fold correctly by inducing chaperones. This multi-level defence system is important for granulosa cells because their survival depends on their ability to stop ROS made by steroidogenic and mitochondrial pathways. Nrf2 activity in granulosa cells is especially important for controlling the metabolism of cysteine and glutathione. Nrf2 causes the gene SLC7A11, which makes the xCT subunit of system Xc^−^, to be transcribed through ARE. Increasing SLC7A11 levels, which is the rate-limiting precursor for glutathione synthesis, makes it easier for cysteine to enter cells [151]. This one regulatory node connects Nrf2 activation to ferroptosis suppression in a mechanistic way because the availability of glutathione affects the enzymatic function of GPX4, which is responsible for detoxifying lipid peroxides. Maintaining the production of glutathione is important for stopping ferroptosis from starting in granulosa cells. These cells are especially vulnerable to lipid peroxidation because they have a lot of PUFA in their membranes, a lot of mitochondria, and a lot of steroidogenic activity.

Keap1’s control of Nrf2 also affects the signalling pathways in mitochondria. ROS made in mitochondria may be able to get into the cytoplasm and hurt Keap1 cysteines. This links mitochondrial failure directly to Nrf2 activation. On the other hand, transcription driven by Nrf2 helps keep mitochondria healthy by lowering oxidative stress, stabilising respiratory complexes, and keeping the integrity of the mitochondrial membrane [152]. Nrf2 detects when mitochondria are in trouble and responds by strengthening the antioxidant machinery needed to bring metabolic balance back to normal. This happens through a feedback loop created by this two-way communication. The Nrf2-Keap1 axis also helps keep iron levels stable in granulosa cells, which is an important factor in ferroptotic susceptibility. When Nrf2 is turned on, it increases the transcription of FTH1 and FTL. This increases the amount of iron that is stored in ferritin nanocages and lowers the amount of iron that is available for Fenton reactions. This function of storing iron becomes even more important during the peri-ovulatory inflammatory cascade, when iron flows and ROS spikes are at their highest. When Nrf2 incorrectly activates ferritin genes, it leads to more hydroxyl radicals, more free Fe^2+^, and the quick start of lipid-peroxidation processes that damage mitochondrial membranes [153].

Nrf2 activation also helps granulosa cells do their job in the endocrine system. Nrf2 keeps redox homeostasis, which protects the structural integrity of steroidogenic enzymes. Many of these enzymes have redox-sensitive residues or need a specific oxidative environment to work. Nrf2 activity stops CYP11A1, CYP19A1, and 3β-HSD from being changed by oxidative stress. It does this by lowering ROS levels and keeping the redox state inside cells stable. The Nrf2-Keap1 system protects luteinisation, the right maturation of follicles, and the production of oestradiol in an indirect way. In granulosa cells, the Nrf2-Keap1 axis is the first, fastest, and most complete antioxidant sensor. It turns oxidative changes into changes that happen all over the genome and keep the structure of mitochondria, stop ferroptosis, keep redox stability, and keep steroidogenic potential. Follicular survival depends on its proper management, and when it doesn’t work right whether because of aging, inflammation, or exposure to toxins it sets the stage for oxidative collapse and follicular atresia.

### 6.2. Nrf2 in Granulosa Cells the Nuclear Translocation, Transcriptional Programs, and Redox Preservation

Once Keap1-induced degradation is stopped, Nrf2 becomes the main regulator of the antioxidant defence in granulosa cells. It starts a chain reaction of transcription that stops oxidative stress while keeping steroidogenic activity, mitochondrial integrity, and the start of ferroptosis [154]. Nrf2’s movement from the cytoplasm to the nucleus is a key molecular event that changes metabolism, redox chemistry, iron regulation, and lipid homeostasis at the genomic level in granulosa cells. When Keap1 releases Nrf2, it builds up in the cytoplasm and quickly moves to the nucleus by exposing hidden nuclear localisation sequences in its Neh1 and Neh5 domains. By heterodimerising with small Maf (musculoaponeurotic fibrosarcoma oncogene) proteins, Nrf2 forms a high-affinity complex that can bind to AREs in the promoter regions of the granulosa cell genome. Nrf2 might use the genomic coding system that these AREs provide to turn on several genes at once that are important for detoxification, redox buffering, iron storage, lipid-peroxide regulation, and keeping mitochondria healthy [155].

Nrf2 initiates the transcription of HO-1 (heme oxygenase-1) within the nucleus. HO-1 is an important enzyme that breaks down heme into biliverdin, free iron, and carbon monoxide. HO-1 protects granulosa cells in two ways: it lowers the levels of redox-active heme, which could otherwise lead to too much ROS production, and biliverdin and carbon monoxide work as antioxidants and anti-inflammatories [156]. HO-1 lets iron out, while Nrf2 tells the body to make a lot of ferritin heavy and light chains. These chains help keep iron in ferritin nanocages. This synchronised induction keeps newly released iron from getting into the labile iron pool, which means it can’t make hydroxyl radicals during follicular oxidative bursts. Nrf2 activates NQO1 (NAD(P)H quinone oxidoreductase 1), an enzyme that stops quinone-mediated redox cycling. This is a major source of reactive oxygen species in mitochondria, especially in tissues that make steroids. NQO1 protects the electron transport chain by keeping quinones stable and not reacting with other molecules. This cuts down on oxidative leakage when energy needs are high [157].

Granulosa cells depend on the glutathione-GPX4 axis to stop ferroptosis, so Nrf2’s control of how glutathione moves around is very important. Nrf2 speeds up the rate-limiting step in making glutathione by turning on GCLC and GCLM. It makes sure that reduced GSH is always made, even when oxidative stress is very high, by making glutathione synthetase and other glutathione reductases transcribe more [81]. This pathway maintains the intracellular GSH/GSSG ratio at a level conducive to follicular survival and supplies the substrate required for GPX4-mediated detoxification of phospholipid hydroperoxides. Nrf2, along with glutathione, controls the thioredoxin system. This is an important antioxidant network that helps keep mitochondria stable, fix protein disulphides, and get rid of peroxides. Nrf2 helps the body make thioredoxin reductase, which helps the body recycle oxidised thioredoxin. This means that granulosa cells can fix proteins that have been damaged by reactive oxygen species and keep the respiratory chain working [158].

Nrf2 plays a big role in keeping mitochondria healthy. Elevated GSH and decreased thioredoxin increase the stability of respiratory complex subunits by lowering electron leakage and maximising oxidative phosphorylation. The creation of superoxide and hydrogen peroxide stays below normal levels, ATP synthesis goes on without a hitch, and the mitochondrial membrane’s potential stays the same [159]. Mitochondrial reactive oxygen species generated in granulosa cells can traverse gap junctions, hindering meiotic spindle formation, chromosomal alignment, and cytoplasmic maturation. Thus, mitochondrial stability safeguards the oocyte. Nrf2 safeguards steroidogenesis concurrently. Oxidative inactivation can happen to redox-sensitive parts of cytochrome P450 enzymes, such as CYP11A1, CYP17A1, and CYP19A1. Nrf2 regulates low levels of reactive oxygen species to stop oxidative interference with steroidogenic pathways and make sure that the right amount of oestradiol is made and luteinisation happens. Nrf2’s important role in keeping the metabolic conditions needed for hormone synthesis is what keeps steroidogenic competence alive, not a side effect [160].

Nrf2 also regulates the balance of lipids in membranes, especially when ferroptotic susceptibility is present. Granulosa-cell membranes have a lot of polyunsaturated fatty acids (PUFAs), which are very likely to oxidise. Nrf2-induced increases in GPX4 activity, ferritin expression, and glutathione availability help stop the buildup of lipid peroxides early on. When Nrf2 isn’t activated enough, lipid peroxidation goes up, which starts the ferroptotic cascade before apoptosis or necrosis. Ultimately, Nrf2 facilitates metabolic synchronisation between granulosa cells and oocytes. Granulosa cells are important for keeping oocytes safe from oxidative damage, giving them antioxidants, and controlling how mitochondrial metabolites move around. When Nrf2 is turned on, granulosa cells will make sure that the redox and metabolic conditions are right for oocyte competence. The absence of Nrf2 disrupts this symbiotic relationship, leading to insufficient oocyte maturation, improper blastocyst development, aberrant spindle formation, and inadequate embryonic genome activation.

### 6.3. Nrf2 Decline in Ovarian Aging, Inflammation, and Environmental Stress

One of the first molecular signs of ovarian ageing is the drop in Nrf2 signalling. This shows a major change in granulosa cells from being resistant to redox stress to being sensitive to oxidative stress and vulnerable to ferroptosis. Ageing leads to a sustained and gradual suppression of Nrf2 activity across various regulatory dimensions, including transcriptional, post-translational, epigenetic, and metabolic pathways, whereas acute oxidative stress rapidly and reversibly activates Nrf2 [160]. This decrease makes it harder for granulosa cells to keep the oocyte alive, get rid of reactive oxygen species, control mitochondrial activity, and keep their steroidogenic competence. As Nrf2 signalling decreases, granulosa cells’ ability to lower oxidative stress also decreases [151]. This makes the follicle more likely to undergo atresia and reproductive decline. Due to alterations in promoter methylation and chromatin accessibility, ageing diminishes the transcriptional level of Nrf2 mRNA synthesis. Research on old ovarian tissue shows that there are more repressive markers like H3K27me3, less H3K4me3 enrichment at the Nrf2 locus, and hypermethylation of ARE-containing promoter regions. These epigenetic changes stop Nrf2 from being expressed, which lowers its baseline levels before oxidative stress starts [161].

The regulatory defect affects the cytoplasmic level, which makes Keap1 more active. Keap1 cysteine residues in juvenile granulosa cells react dynamically to ROS through reversible oxidative changes. However, as Keap1 ages, its oxidation state and conformation change, making it more resistant to inactivation and better at sequestering Nrf2 [162]. This change happens because of long-term low-level oxidative stress, which is a common sign of ovarian ageing. This stress paradoxically leads to a Keap1-dominant inhibitory state. Increased Keap1 activity speeds up the breakdown of Nrf2, which lowers its presence in the nucleus and makes it harder for ARE-mediated transcription to happen. Mitochondrial dysfunction makes the decline of Nrf2 with age much worse. Granulosa cells are under constant oxidative stress because they don’t have enough Nrf2-mediated protection [163]. This is because mitochondria build up DNA damage, lower membrane potential, and make more reactive oxygen species. Malfunctioning mitochondria release ROS into the cytosol all the time. This happens because mitophagy isn’t working properly, which is mostly because of Nrf2. This sets off a harmful cycle: long-term exposure overloads the sensory system and makes the Keap1-Nrf2 axis less responsive, even though mitochondrial ROS is thought to activate Nrf2 [49]. Even with low activation, Nrf2’s ability to do its job is hurt by the fact that NADPH levels drop in older cells, which makes it harder for antioxidants to be made again.

This drop in Nrf2 has big and complicated effects. The first effect is that the body’s stores of glutathione run low. Granulosa cells cannot maintain the necessary glutathione levels for GPX4 activity due to reduced transcription of GCLC, GCLM, and glutathione synthetase [164]. Granulosa cells are susceptible to ferroptosis owing to the resultant impairment of the glutathione-GPX4 axis, particularly under conditions of heightened oxidative stress associated with steroidogenesis. When Nrf2-mediated cystine import through SLC7A11 is inadequate, glutathione synthesis is impaired, lipid peroxides accumulate, and ferroptotic signalling prevails. Another effect is that the thioredoxin and peroxiredoxin systems stop working and recycling because they rely on Nrf2 transcription [60]. As these systems break down, protein disulphide repair slows down, oxidative damage stays in subunits of mitochondrial complexes I and III, electron leakage goes up, and the production of ROS goes up quickly. More ROS in the cytoplasm and mitochondria make mitochondrial membranes even less stable, stop steroidogenic enzymes from working, and speed up lipid peroxidation.

Nrf2 reduction affects iron metabolism, which is an important part of ferroptosis. Because HO-1 regulation and the transcription of ferritin heavy and light chains (FTH1, FTL) are both lower, free iron builds up in the cytosol and mitochondria. Senescent ovaries have bigger pools of labile iron, more Fe^2+^ available, and more Fenton activity [165]. These things almost guarantee that lipid ROS will be made and ferroptosis will happen. Inflammatory ovarian conditions like endometriosis lower Nrf2 levels more quickly. TNF-α, IL-6, and IL-1β are cytokines that stop Nrf2 transcription, raise Keap1 expression, and make reactive oxygen species that quickly outstrip the antioxidant abilities of granulosa cells [166]. Iron-laden endometriotic lesions add too much free iron to follicular fluid, which makes oxidative stress worse and the Nrf2 response even weaker. Granulosa cells in endometriosis exhibit significant oxidative dysfunction, diminished steroidogenic potential, compromised oocyte support, and increased ferroptotic markers.

Metabolic dysfunction in PCOS also stops Nrf2 signalling by causing lipotoxicity, high insulin levels, advanced glycation end products, and long-term low-grade inflammation. Granulosa cells in PCOS show early signs of being susceptible to ferroptosis, such as broken mitochondria, a lower mitochondrial membrane potential, less Nrf2 activity, and higher lipid peroxidation [167]. These irregularities elucidate the reason that, despite their prevalence, PCOS follicles often generate oocytes with compromised developmental competence. Pollution in the environment also makes Nrf2 suppression worse. Exposing cells to bisphenol A, phthalates, cadmium, arsenic, and airborne particles changes the cysteines in Keap1, encourages oxidative DNA damage, and affects the Keap1-Nrf2 interaction [168]. These harmful substances speed up follicular atresia even in young people by lowering ARE activation, raising iron-mediated lipid oxidation, and lowering glutathione production. Table 3 lists the main Nrf2-regulated targets that are important for maintaining redox homeostasis in the ovaries. This gives a full picture of how Nrf2 controls antioxidant and anti-ferroptotic defences in granulosa cells.

### 6.4. Nrf2 as a Master Anti-Ferroptotic Regulator in the Follicle

Nrf2 is the most comprehensive mechanism for safeguarding the ovarian follicle from ferroptosis, extending beyond mere regulation of antioxidants in granulosa cells. Granulosa cells possess many mitochondria, abundant PUFA-rich membranes, and generate ROS that facilitate steroidogenesis, making ferroptosis a significant vulnerability [60]. Ferroptosis occurs when iron induces lipid peroxidation and the failure of GPX4. Iron overload, glutathione depletion, GPX4 inactivation, lipid-peroxide buildup, and mitochondrial destabilisation are upstream initiators of ferroptosis that Nrf2 mitigates in this biochemical setting by coordinating a multifaceted defensive mechanism [60,169]. Thus, its reduction creates the ideal molecular environment for follicular atresia and ferroptotic cell death. Nrf2’s capacity to inhibit ferroptosis derives from its regulation of cysteine import and glutathione synthesis, both essential for GPX4 functionality. Nrf2 promotes cystine absorption by system Xc^−^ by activating SLC7A11 transcription, thus supplying the necessary substrate for glutathione production via GCLC and GCLM. In the absence of Nrf2, this supply chain ceases to function: glutathione levels diminish, cystine transport is inhibited, and GPX4 loses its electron donor [170]. Lipid peroxides may accumulate in mitochondrial and plasma membranes due to the inability to eliminate phospholipid hydroperoxides. This initiates the ferroptotic cascade. Nrf2 safeguards granulosa cells by maintaining the whole metabolic pathway essential for GPX4 functionality, rather than only a single component.

The control of iron homeostasis by Nrf2 is significant since it influences vulnerability to ferroptosis. Nrf2 enhances the cell’s iron-sequestration mechanism by activating ferritin heavy and light chains (FTH1 and FTL). This diminishes the cytosolic labile iron pool (LIP). Ferritin securely sequesters Fe^3+^ to prevent its conversion to Fe^2+^, the reactive form involved in the Fenton reaction that generates hydroxyl radicals. These are very active initiators of lipid peroxidation [165]. Nrf2 meticulously regulates the release of iron by modulating the functionality of iron-handling proteins such as ferroportin and HO-1. The Nrf2-mediated sequestration is crucial in granulosa cells, which contain several iron-sulfur clusters in mitochondria, with iron flux escalating during steroidogenesis and ovulatory inflammation. In its absence, free iron accumulates, mitochondrial Fenton reactions accelerate, and lipid reactive oxygen species increase, constituting the first biochemical indicator of ferroptosis [153].

Nrf2 mitigates lipid peroxidation, contributing to its anti-ferroptotic function, by regulating the production of antioxidant genes and preserving membrane integrity. Nrf2-mediated transcription elevates the concentrations of glutathione peroxidases, peroxiredoxins, oxidoreductases, NQO1, and phase II detoxification enzymes. These enzymes inhibit the propagation of lipid radical chains and neutralise lipid peroxides. Nrf2 prevents PUFA-phospholipids in mitochondrial and endoplasmic reticulum membranes from reaching the oxidation threshold that initiates ferroptotic execution by enhancing GPX4 transcription and indirectly safeguarding it via the maintenance of elevated glutathione levels [171].

Granulosa cells possess membranes rich in phosphatidylethanolamine species including AA and adrenic acid (AdA), which serve as optimal substrates for ferroptotic peroxidation. This makes the regulation of membrane lipid stability more critical. Granulosa cells mitigate the harmful oxidation of PUFA-rich phospholipids, even under significant oxidative stress, by activating enzymes that neutralise peroxyl radicals via Nrf2. Nrf2’s involvement in preserving mitochondrial resilience, a critical component of ferroptosis resistance, is likely its most notable function [172]. Nrf2 facilitates mitochondrial detoxification by maintaining thioredoxin reductase activity, regenerating NADPH, and safeguarding respiratory chain proteins from oxidative inactivation. This results in reduced electron leakage, less ROS production, and a steady mitochondrial membrane potential. When Nrf2 is functional, mitochondria combat alterations occurring during ferroptosis, including shrinkage, cristae collapse, and hyper-condensation. However, as Nrf2 levels diminish, iron escapes from iron-sulfur clusters, defective mitochondria accumulate, and mitochondrial lipid peroxidation increases, rendering the avoidance of ferroptosis unfeasible [173].

Nrf2 further inhibits ferroptosis by modulating proteostasis and facilitating communication between the endoplasmic reticulum and mitochondria. Nrf2 inhibits the accumulation of ROS induced by ER stress and maintains the phospholipid remodelling pathways that modulate ferroptotic sensitivity via the synthesis of chaperones, redox enzymes, and lipid-detoxifying proteins [174]. The stabilisation of ER–mitochondrial contact sites inhibits the pathogenic lipid remodelling that occurs prior to ferroptosis by maintaining equilibrium in calcium and lipid fluxes. The activation of Nrf2 is the primary determinant of the response of granulosa cells to stress induced by chemicals, metabolic changes, or inflammation. When Nrf2 is sufficiently active, it regulates cystine import, glutathione production, iron sequestration, GPX4 activity, and mitochondrial stability [175]. Granulosa cells facilitate oocyte maturation, sustain steroidogenesis, and prevent ferroptotic collapse under significant increases in ROS. The multi-layered anti-ferroptotic barrier deteriorates as Nrf2 levels decline, which may occur due to ageing, endometriosis, polycystic ovary syndrome, environmental pollutants, or ovarian chemotherapy. Iron accumulates, glutathione levels decline, GPX4 becomes inactive, lipid peroxidation intensifies, and mitochondrial membranes degenerate. The irreversible ferroptotic cell death in this cascade leads to follicular atresia and accelerated ovarian ageing. Nrf2 serves as the primary anti-ferroptotic regulator for the follicle. A singular molecular defence program encompasses redox sensing, iron regulation, glutathione metabolism, lipid-peroxide detoxification, mitochondrial preservation, steroidogenic compatibility, and oocyte safeguarding. The decrease is a mechanistic catalyst that induces ferroptosis and diminishes reproductive capacity, rather than just being a natural aspect of ageing [176].

## 7. Crosstalk Between *SIRT1* and Nrf2 in Anti-Ferroptotic Defense

The functional convergence of *SIRT1* and Nrf2 constitutes the primary survival circuitry of granulosa cells. This integrated axis of antioxidants, metabolism, and anti-ferroptosis dictates whether oxidative stress remains physiologically adaptable or escalates into severe cellular damage. *SIRT1* and Nrf2 are often regarded as distinct regulatory systems one controlled by transcription and the ARE, the other by metabolism and deacetylation yet increasing mechanistic evidence indicates that both pathways function collaboratively as an interconnected network. Their profound, mutual communication is essential for the persistence of follicles. Granulosa cells, characterised by the structural fragility of mitochondria, elevated polyunsaturated fatty acid content in cellular membranes, and heightened redox activity during steroidogenesis, represent the only location in the ovary where this partnership is especially critical for ferroptosis sensitivity.

### 7.1. SIRT1-Driven Activation of Nrf2 Signaling

*SIRT1* has a complicated effect on Nrf2 signalling in granulosa cells. It changes both the biochemical quality of the antioxidant response and how long and how much Nrf2 is activated. The connection between *SIRT1* and Nrf2 is not random or linear. Instead, it is a highly integrated axis that connects the master antioxidant transcriptional program to metabolic sensing, mitochondrial redox flux, and chromatin remodelling [177]. To prevent redox imbalance, ferroptotic priming, and irreversible follicular degeneration in granulosa cells, it is essential to modulate Nrf2 in a *SIRT1*-dependent manner. Steroidogenesis, mitochondrial respiration, and FSH signalling all make oxidative byproducts all the time in these cells. *SIRT1* directly changes Nrf2 at the molecular level by targeting lysine deacetylation, which is a process that happens after translation that makes Nrf2 more stable, binds to DNA better, and improves transcriptional accuracy [178]. There are regulatory lysine clusters in Nrf2’s Neh1, Neh4, and Neh5 domains. These are the parts that stick to ARE and bring in transcriptional coactivators. When acetylated, these lysines make Nrf2 less able to bind to ARE motifs and stop it from interacting with them in a stable way. By removing acetyl groups from these residues, *SIRT1* increases chromatin occupancy. This improves Nrf2’s transcriptional interface and draws in more Maf proteins and RNA polymerase II. For granulosa cells to stay alive, they need to be deacetylated. This means that the cells are going from a low-output antioxidant state to a high-efficiency transcriptional program that protects cells [179].

*SIRT1* not only directly changes Nrf2, but it also has a big effect on the Keap1-Cul3 sensor complex, which sets the level at which Nrf2 can be activated. The oxidation state of key cysteine residues like Cys151, Cys273, and Cys288, which can be changed back and forth by oxidative modifications that control Nrf2 release, decides how Keap1 is controlled. *SIRT1* alters these cysteine-sensitive switches by regulating the production of ROS in the mitochondria [180]. When *SIRT1* is working well, there isn’t much electron leakage, basal ROS levels stay normal, and mitochondrial electron transport works well. This controlled redox environment lets Nrf2 be activated in a sensitive and exact way when oxidative stress rises. It also stops Keap1 from being oxidised in the wrong way [177]. On the other hand, when *SIRT1* levels drop, mitochondrial ROS suddenly rise. This happens when someone gets older, has metabolic problems, or gets hurt by chemicals. This makes Keap1 oxidise in a bad way, Nrf2 to be released in a way that isn’t always clear, and the body’s ability to fight off free radicals to become less effective or worn out. This is how *SIRT1* keeps the Nrf2 activation switch safe.

*SIRT1* also changes how Nrf2 is turned on by changing how metabolism works. By promoting the deacetylation of PGC-1α, *SIRT1* makes oxidative phosphorylation, fatty acid β-oxidation, and mitochondrial biogenesis work better. PGC-1α improves how mitochondria work, which lowers the amount of ROS that enters the cytoplasm and the background “noise” that would normally cause Nrf2 to activate too soon [181]. This metabolic stabilisation allows granulosa cells to maintain a pristine redox environment, ensuring that Nrf2 activation occurs solely in response to genuine oxidative threats, including ovulatory inflammation, gonadotropin-induced steroidogenesis, or exposure to environmental toxins. *SIRT1* acts as a redox gatekeeper in this setup, stopping long-term, low-level Nrf2 activation. If this didn’t happen, antioxidant stores would run out and responsiveness would go down [182].

FOXO3 is a transcription factor that helps cells deal with oxidative stress, and *SIRT1* controls it. This gives *SIRT1* more power. FOXO3 is turned on by *SIRT1*-mediated deacetylation, which increases the transcription of antioxidant genes like SOD2 and catalase. This extra ability to neutralise ROS stops Keap1 cysteines from oxidising when they shouldn’t and changes how much Nrf2 is released by lowering the amount of peroxide that builds up in the cytosol [183]. FOXO3 and Nrf2 work together in granulosa cells to create a double layer of protection against free radicals. Nrf2 gives a lot of antioxidant support that can be turned on and off, while FOXO3 keeps detoxifying mitochondrial ROS all the time. *SIRT1* also affects Nrf2 by keeping the levels of NAD^+^ steady. For Nrf2 to work, the pools of NADPH and NAD^+^ must be intact so that glutathione and thioredoxin can be recycled [184]. *SIRT1* controls NAMPT, AMPK, and the flow of energy in the mitochondria to keep NAD^+^ levels high and stop them from dropping when PARP1 is too active under oxidative stress. PARP1 uses NAD^+^ for poly-ADP ribosylation when DNA is badly damaged or someone has been exposed to a lot of chemicals. This could take away *SIRT1*’s important cofactor. *SIRT1* indirectly stops PARP1 from being too active, which keeps NAD^+^ levels stable and lets Nrf2 stay active for longer by keeping redox stability and lowering DNA breaks [185].

### 7.2. Preservation of GPX4 as the Core Anti-Ferroptotic Shield in Granulosa Cells

The last biochemical step that defines whether granulosa cells can resist or perish from ferroptosis is the maintenance of GPX4. GPX4 (glutathione peroxidase 4) is a unique antioxidant enzyme since it can directly break down phospholipid hydroperoxides that are lodged in biological membranes, such the mitochondria and endoplasmic reticulum, where ferroptotic lesions initially arise [186]. GPX4 targets oxidised phosphatidylethanolamines and phosphatidylcholines inside lipid bilayers, neutralising lipid radicals before they propagate into chain reactions that destabilise membranes, unlike typical peroxidases that detoxify freely soluble peroxides. GPX4 is the most significant factor that impacts survival, redox equilibrium, and the health of the follicles. This is because granulosa cells have a lot of PUFA-containing phospholipids, a lot of mitochondria, and operate in regions where steroidogenic ROS is constantly being generated [187]. The *SIRT1*-Nrf2 axis directly interacts with GPX4 at the transcriptional, metabolic, post-translational, and redox-regulatory levels to form the strongest anti-ferroptotic mechanism in the follicle.

Nrf2 regulates the transcription of genes that govern the synthesis and recycling of glutathione, thereby ensuring the availability of GPX4, which requires glutathione as its electron donor for functionality. Nrf2 stimulates SLC7A11, which permits cystine to be transported into the body via the system Xc^−^ transporter [188]. Cystine rapidly converts into cysteine, which is required to generate glutathione. Nrf2 then creates the catalytic and modifier subunits of glutamate cysteine ligase, GCLC and GCLM, which are responsible for the rate-limiting step in the manufacture of glutathione. Nrf2 ensures the continuous production of reduced GSH, essential for GPX4 to convert membrane-bound hydroperoxides into non-toxic alcohols, via coordinating the activation of glutathione synthetase and glutathione reductase. GPX4 loses its capacity to catalyse processes when there is no carefully regulated supply of glutathione, even if its protein levels remain the same. Nrf2 safeguards the biochemical functionality of GPX4 under oxidative stress and its capacity for RNA synthesis [189].

*SIRT1* enhances this defence by preserving the metabolic conditions necessary for GPX4 stability and functionality. *SIRT1* inhibits the generation of mitochondrial superoxide, decreases electron leakage, and helps mitochondrial oxidative phosphorylation perform better via deacetylating and activating PGC-1α [190]. This decline in ROS reduces the oxidative strain on GPX4 by decreasing phospholipid oxidation, which protects GPX4 from being inactivated too soon. Moreover, during steroidogenic surges, *SIRT1*-mediated AMPK activation suppresses redox collapse and protects NADPH stores, the required redox currency for glutathione recycling. *SIRT1* maintains the metabolic environment correct for GPX4 turnover to keep occurring since GPX4 requires GSH and GSH needs enough NADPH to be produced.

*SIRT1* preserves GPX4 functionality by inhibiting p53-induced suppression of SLC7A11, a critical but often neglected mechanism. In the presence of genotoxic or metabolic stress, P53 inhibits SLC7A11 transcription, significantly reducing cystine import and initiating ferroptotic priming. *SIRT1* reduces the transcriptional activity of p53 by deacetylating it. This prevents the silencing of SLC7A11 [191]. This regulatory interaction positions *SIRT1* as a molecular buffer that facilitates the influx of cystine under challenging conditions, hence ensuring the continuous synthesis of glutathione and safeguarding GPX4 function. This pathway is crucial in granulosa cells under stress from chemotherapy, inflammation, or ageing: in the absence of *SIRT1*, p53 inhibits SLC7A11, leading to decreased glutathione levels, impaired GPX4 function, and the occurrence of ferroptosis.

Selenium must also be incorporated into the active site of GPX4’s selenocysteine residue. *SIRT1* facilitates this process indirectly by reducing oxidative stress that may otherwise oxidise selenocysteine or induce selective degradation of GPX4 [192]. Nrf2 promotes the stability of selenoproteins by preserving redox equilibrium in the endoplasmic reticulum, where GPX4 undergoes folding and maturation. The *SIRT1*-Nrf2 axis collaborates to ensure the correct folding, enzymatic activity, and protection from oxidative degradation of the GPX4 protein. Due to its significance, GPX4 is very susceptible to occurrences prevalent in endometriosis, PCOS, ovarian ageing, and chemotoxic damage [193]. With advancing age, *SIRT1* activity diminishes, resulting in NAD^+^ depletion, mitochondrial malfunction, and chronic inflammation. This complicates the body’s synthesis of glutathione and NADPH. When Nrf2 signalling is compromised due to impaired Keap1 sensing, epigenetic silencing, or the presence of toxins, GPX4 transcription is inhibited, resulting in insufficient glutathione synthesis machinery.

### 7.3. The Mitochondrial ROS-Lipid Metabolism Axis as a Central Determinant of Ferroptotic Vulnerability in Granulosa Cells

The mitochondrial ROS lipid metabolism axis is the paramount cellular system influencing the sensitivity of granulosa cells to ferroptosis. Mitochondria generate the majority of the reactive oxygen species present in the body. They also maintain the stability of iron-sulfur clusters and exhibit the initial structural alterations that occur during ferroptosis [71]. Their membranes are rich in polyunsaturated phospholipids, such as AdA and AA, which are conducive to lipid peroxidation. These characteristics render granulosa cells highly susceptible to oxidative stress, a condition characterised by the breakdown of lipids and the production of ROS in the mitochondria. The *SIRT1*-Nrf2 axis regulates this intersection by inhibiting both enzymatic and non-enzymatic processes that convert PUFA-rich phospholipids into ferroptotic substrates, while maintaining mitochondrial stability [194].

The mitochondria in granulosa cells must exert considerable effort. They govern the transport of cholesterol across the mitochondrial membrane, supply ATP for steroidogenic enzyme function, and orchestrate the synthesis of NADPH, crucial for glutathione recycling [195]. During steroidogenesis, electron leakage at complexes I and III increases due to accelerated mitochondrial electron transport, particularly in the presence of gonadotropin. Insufficient detoxification results in the conversion of molecular oxygen into superoxide by released electrons, which rapidly transforms into hydrogen peroxide [196]. In the absence of neutralisation by Nrf2-regulated systems such as GPX family members, peroxiredoxins, and glutathione peroxidases, hydrogen peroxide engages in Fenton chemistry with mitochondrial Fe^2+^, generating hydroxyl radicals that target adjacent PUFA-phospholipids. Mitochondrial reactive oxygen species are not merely detrimental byproducts; they are the catalysts of ferroptosis. Ferroptosis is lethal only when lipid peroxidation proliferates uncontrollably across mitochondrial and endoplasmic reticulum membranes.

*SIRT1* participates early in this process by enhancing mitochondrial function and reducing ROS production. *SIRT1* enhances fatty acid catabolism, respiratory chain complex assembly, and mitochondrial biogenesis by deacetylating PGC-1α [197]. These effects diminish superoxide production at its origin, maintain membrane potential stability, and prevent electron leakage. *SIRT1* enhances mitophagy by deacetylating pathways associated with FOXO3 and PINK1. This ensures the removal of damaged mitochondria that release reactive oxygen species, rather than retaining them in the cytoplasm as oxidative catalysts. In the absence of *SIRT1*, defective mitochondria accumulate, leading to the disintegration of their iron-sulfur clusters, which releases free iron and initiates a self-propagating amplification of ROS. This biochemical condition accelerates ferroptotic priming.

Nrf2 collaborates with *SIRT1* to eliminate mitochondrial reactive oxygen species prior to the initiation of lipid peroxidation. Nrf2 induces the transcription of SOD2, thioredoxin reductase, peroxiredoxin 3, and components of glutathione metabolism, therefore establishing a multifaceted enzymatic barrier around mitochondrial membranes [198]. This barrier prevents superoxide and hydrogen peroxide from forming lipid-bound hydroperoxides. Mitochondria rely mostly on Nrf2-regulated detoxification mechanisms for redox protection because of the little catalase activity present in the cytoplasm. The first and most fatal ferroptotic substrates, AA-PE and AdA-PE molecules, experience fast oxidation when mitochondrial ROS evade detoxification owing to impaired Nrf2 signalling, as seen in ageing, endometriosis, inflammation, or exposure to toxicants.

Lipid metabolism enhances this process in granulosa cells. Their membranes have a high concentration of polyunsaturated phospholipids, particularly phosphatidylethanolamines with cis-double bonds that are very susceptible to oxidation. Under oxidative and inflammatory circumstances, the enzymes ACSL4 and LPCAT3 intensify their activity to incorporate PUFA into these phospholipids [71]. As oxidative stress increases, these enzymes modify membranes by incorporating PUFA-rich lipids, therefore preparing the biochemical milieu for ferroptosis. Nrf2 often mitigates this vulnerability by decreasing iron availability and promoting GPX4-mediated detoxification of lipid hydroperoxides, hence restricting Fenton-induced peroxidation. However, when Nrf2 levels diminish, ACSL4-mediated PUFA loading persists, converting membrane lipids into oxidisable substrates that can initiate mitochondrial ROS production [199].

The endoplasmic MAMs serves as a crucial site for amplification in ferroptosis. These contact sites facilitate redox signalling, calcium flux, and lipid transfer. Calcium release from the endoplasmic reticulum into mitochondria under oxidative conditions accelerates lipid peroxide formation, elevates reactive oxygen species levels, and induces mitochondrial depolarization [200]. *SIRT1* stabilises MAMs by maintaining phospholipid equilibrium, ensuring proper calcium buffering, and reducing endoplasmic reticulum stress through the deacetylation of unfolded protein response regulators. Nrf2 eliminates peroxides from the body and enhances the mechanisms responsible for protein folding to alleviate stress on the endoplasmic reticulum. When both systems malfunction, MAMs transform into sites of rapid lipid-peroxide amplification, resulting in the morphological characteristic of ferroptosis, namely the swift disintegration of mitochondrial cristae [201].

Iron metabolism is intricately linked to this axis. Mitochondria are abundant in iron-sulfur clusters and heme-containing enzymes. Oxidative stress degrades these clusters, resulting in the release of Fe^2+^ into the mitochondrial matrix. The combination of free iron and hydrogen peroxide generates hydroxyl radicals that target adjacent PUFA-phospholipids [202]. *SIRT1* inhibits this process by reducing ROS flux and enhancing the stability of mitochondrial enzymes. Nrf2 enhances this process by promoting ferritin synthesis and regulating heme turnover through HO-1. If either system deteriorates, lipid peroxidation accelerates, mitochondrial iron accumulates, and the ferroptotic cascade becomes irreversible. The interconnected molecular events indicate that the mitochondrial ROS-lipid metabolism axis is the mechanistic core of ferroptotic susceptibility in granulosa cells [203]. *SIRT1* and Nrf2 collaborate to inhibit the lipid-peroxide cascade that occurs during ferroptosis. *SIRT1* diminishes the generation of reactive oxygen species in mitochondria and accelerates mitochondrial repair. Nrf2, conversely, eliminates ROS and safeguards membranes rich in PUFA. Upon the disruption of this axis, the granulosa cell embarks on an irreversible trajectory towards ferroptotic demise, follicular atresia, and reproductive deterioration. This may occur because to ageing, oxidative stress, inflammation, metabolic malfunction, or chemotherapy.

### 7.4. An Integrative Conceptual Model, Coordination of FOXO3, PGC-1α, AMPK, Iron Flux, and Ferritinophagy in SIRT1-Nrf2-Mediated Anti-Ferroptotic Protection

Granulosa cells exhibit resistance to ferroptosis via many non-linear pathways. Rather, a sophisticated, intricately coordinated molecular network exists in which *SIRT1* and Nrf2 function as principal regulators. Their collective effects influence several biochemical processes, including cellular metabolism, mitochondrial dynamics, iron homeostasis, lipid remodelling, and antioxidant capability [145]. These processes establish a cohesive survival framework capable of enduring the extremely oxidative and inflammatory surroundings of the ovarian follicle. This section integrates all interacting modules into a singular conceptual model to illustrate how FOXO3, PGC-1α, AMPK, iron flux, and ferritinophagy collaborate with the *SIRT1*-Nrf2 axis to determine the destiny of granulosa cells.

The first component of the network is *SIRT1*, a precise metabolic sensor that functions optimally in the presence of sufficient NAD^+^ inside the cell. Granulosa cells have alterations in NAD^+^ levels when subjected to metabolic stress from gonadotropin stimulation, mitochondrial hyperactivity, or inflammatory cytokines [204]. *SIRT1* is the primary regulator that converts these alterations into adaptive responses. *SIRT1* deacetylates FOXO3, enhancing its propensity to transcribe antioxidant genes such as SOD2 and catalase, along with genes that regulate mitophagy. This maintains the mitochondrial membrane potential, enhances the stability of electron transport, and directly reduces the production of reactive oxygen species in the mitochondria [205]. *FOXO3* serves as the primary defence mechanism by reducing intracellular ROS to prevent the initiation of lipid peroxidation. *SIRT1* promotes PGC-1α, which regulates oxidative phosphorylation and mitochondrial biogenesis. PGC-1α ensures the formation of new mitochondria with complete respiratory complexes, robust membrane potential, and optimal cristae architecture. Revitalising the mitochondrial network mitigates the deterioration of iron-sulfur clusters inside mitochondria and prevents electron leakage. The renewal process is crucial in granulosa cells, where oxidative stress and ageing often impair mitochondria, diminishing respiration efficiency and increasing the likelihood of ferroptosis. PGC-1α confers resistance to the oxidative amplification loops that initiate ferroptosis in mitochondria by this mechanism [206].

AMPK, via activating *SIRT1* and PGC-1α, enhances this adaptive metabolic program. During energetic stress, such as low ATP levels during ovulatory inflammation or elevated steroidogenic demand, AMPK phosphorylates PGC-1α, facilitating NAD^+^ regeneration. This sustains *SIRT1* activity [207]. AMPK-mediated fatty acid β-oxidation prevents excessive incorporation of oxidisable PUFA species into membrane phospholipids by reducing the levels of PUFA in the cytosol and mitochondria. AMPK diminishes the biochemical capacity for ferroptotic lipid peroxidation by reducing the availability of polyunsaturated fatty acid substrates. AMPK, *SIRT1*, and PGC-1α collaborate to maintain mitochondrial energetics and regulate redox homeostasis. Nrf2 serves as the transcriptional regulator of antioxidant and anti-ferroptotic gene expression, concurrently initiating both processes. Nrf2-mediated ARE activation regulates the synthesis of glutathione, thioredoxin, NADPH-generating enzymes, peroxiredoxins, and GPX4. This establishes the metabolic framework that prevents the accumulation of lipid hydroperoxide directly. Nrf2 regulates the synthesis of ferroportin, HO-1, and the heavy and light chains of ferritin (FTH1 and FTL) [208]. These three proteins collaborate to regulate the intracellular distribution of iron. The capacity of ferritin to sequester iron is crucial for inhibiting iron-mediated Fenton reactions in mitochondria, which lead to lipid peroxidation.

The relationship between iron metabolism and mitochondrial reactive oxygen species is a critical juncture in this concept. Oxidative stress damages iron-sulfur clusters in the electron transport chain, resulting in the release of Fe^2+^ into the mitochondrial matrix. This free iron rapidly interacts with hydrogen peroxide to generate hydroxyl radicals, initiating the process of PUFA-phospholipid peroxidation [209]. Nrf2 sequesters free iron inside ferritin nanocages. However, *SIRT1* mitigates this detrimental process by reducing the production of ROS by mitochondria. They collaborate to inhibit the biochemical mechanism that characterises ferroptosis and maintain stable mitochondrial iron concentrations. A detrimental process that occurs as the *SIRT1*-Nrf2 axis diminishes is ferritinophagy, the degradation of ferritin by NCOA4. Ferritinophagy often liberates iron in a regulated manner to sustain metabolic functions. However, when ferritinophagy is too active, it elevates the labile iron pool in the presence of oxidative or metabolic stress. This introduces redox-active Fe^2+^ to mitochondria and accelerates lipid peroxidation. Nrf2 inhibits ferritin degradation by enhancing ferritin transcription. *SIRT1*, conversely, inhibits aberrant ferritinophagy by reducing ROS levels and maintaining mitochondrial functionality. Ferroptosis is inevitable if either mechanism malfunctions, since ferritin is destroyed more swiftly than it is produced, leading to iron buildup [165].

This conceptual paradigm also encompasses the contact between the endoplasmic reticulum and the mitochondria. Oxidative stress disrupts the calcium exchange between the endoplasmic reticulum and mitochondria. This prompts the mitochondria to produce more ROS and facilitates lipid remodelling. Nrf2 eliminates peroxides originating from the endoplasmic reticulum and facilitates proper protein folding [176]. *SIRT1*, conversely, mitigates endoplasmic reticulum stress by regulating the unfolded protein response. When combined, they maintain communication between the endoplasmic reticulum and mitochondria, preventing calcium from exacerbating oxidative stress and lipid peroxidation. When these components are assembled, the resulting network may be conceptualised as an anti-ferroptotic circuit with several connections. Ferritin sequesters iron, Nrf2 detoxifies oxidised lipids and proteins, AMPK maintains energy homeostasis, FOXO3 reduces reactive oxygen species, PGC-1α regenerates damaged mitochondria, and *SIRT1* integrates these systems cohesively. This integrated framework enables granulosa cells to manage the elevated metabolic, oxidative, and hormonal requirements of follicular physiology. When any node in this network fails due to age-related NAD^+^ depletion, chronic inflammation, metabolic dysfunction, environmental pollutants, or chemotherapy, the whole circuit becomes unstable. This results in a rapid transition to ferroptotic priming, marked by iron accumulation, mitochondrial dysfunction, oxidation of PUFA-phospholipids, GPX4 depletion, and ultimately, hair follicle demise.

## 8. Therapeutic Insights and Translational Perspectives

The discovery that ferroptosis is a key factor in the death of granulosa cells opens up a whole new way to treat reproductive medicine. This approach entails the active restoration of the molecular systems that protect the follicle from iron-induced lipid peroxidation, rather than merely mitigating oxidative stress. Our current understanding of the *SIRT1*-Nrf2 axis indicates that ovarian ageing is a gradual deterioration of the biochemical framework responsible for antioxidant capacity, mitochondrial stability, lipid integrity, and metabolic flexibility, rather than a mere accumulation of damage. The most promising ways to keep the ovaries working, improve the quality of oocytes, and extend the reproductive lifespan are therapies that can rebuild this infrastructure. These include boosting *SIRT1*, activating Nrf2, stabilising GPX4, controlling iron flow, or making mitochondria more resilient.

Granulosa-cell ferroptosis can explain many clinical phenomena, such as decreased ovarian reserve, decreased blastocyst formation, increased aneuploidy, failed implantation, low oocyte competence in PCOS, follicular burnout in endometriosis, and chemotherapy-induced premature ovarian insufficiency. Mitochondrial dysfunction, glutathione depletion, iron overload, inflammatory cytokine accumulation, and impaired *SIRT1* or Nrf2 signalling are all signs of ferroptotic vulnerability. Each of these conditions has some of these signs, but not all of them. So, treatments that boost anti-ferroptotic pathways not only help with symptoms, but they also restore molecules. At the molecular level, activating *SIRT1* with drugs is a very effective strategy. Resveratrol, NR, NMN, and synthetic *SIRT1* activators are some of the substances that increase the availability of NAD^+^ and directly improve the function of the *SIRT1* enzyme. These agents enhance mitochondrial function, augment antioxidant defence, stabilise GPX4, inhibit p53-mediated repression of SLC7A11, and restore metabolic-redox homeostasis via *SIRT1*-mediated deacetylation of PGC-1α, FOXO3, and Nrf2. Taking resveratrol has been linked to more MII oocytes, fewer oxidative biomarkers in follicular fluid, a better mitochondrial membrane potential in granulosa cells, and better blastocyst formation in human IVF populations. These findings suggest that one of the initial “ovarian rejuvenation” methods grounded in mechanistic biology rather than empirical supplementation could be *SIRT1* activation.

Nrf2 activation is another important part of treatment that works alongside *SIRT1* targeting. Sulforaphane, curcumin analogues, lipoic acid, and certain polyphenols are natural Nrf2 activators that enhance Nrf2’s nuclear translocation and stimulate the transcription of GCLC, GCLM, SLC7A11, HO-1, NQO1, FTH1, and GPX4. These changes make peroxides less toxic, raise the levels of glutathione, speed up the cycling of thioredoxin, and trap free iron. When Nrf2 is activated, it raises GSH levels, restores steroidogenic activity, and improves mitochondrial respiration in granulosa cells taken from older IVF patients. The Nrf2 pathway also supports the use of precision medicine in chemotherapy-induced ovarian failure. Nrf2 activators may stop the lipid peroxidation caused by iron that destroys primordial follicles that have been exposed to alkylating agents by keeping iron levels stable and stopping ferritinophagy. The combined effects of activating *SIRT1* and Nrf2 seem to be stronger than the effects of either pathway on its own. Nrf2 keeps the NADPH and redox balance that *SIRT1* needs to work, and *SIRT1* boosts Nrf2’s transcriptional potency by deacetylating it. This positive feedback loop strengthens the anti-ferroptotic state by making it easier for mitochondria to get rid of ROS, keeping GPX4 stable, and keeping cystine import going. From a translational point of view, dual-targeted interventions are the next big thing in fertility treatments. In theory, *SIRT1*-Nrf2 co-activation could revert an ovary from a ferroptosis-susceptible condition to a safeguarded, metabolically adaptable phenotype conducive to the formation of high-quality oocytes.

Another new way to treat ferroptosis is to stop it directly. Iron chelators such as deferoxamine and deferiprone, along with molecules like ferrostatin-1 and liproxstatin-1, directly impede lipid peroxidation or diminish the labile iron pool. Many preclinical ovarian models demonstrate that ferroptosis inhibitors sustain hormone production, preserve follicular architecture, and avert granulosa cell apoptosis, although they have not yet been implemented clinically in reproductive medicine. Ferroptosis inhibitors can be administered simultaneously with gonadotoxic chemotherapy, presenting an appealing strategy for preserving fertility. Preventing ferroptosis also works with mitochondrial therapies like CoQ10, melatonin, and antioxidants that target mitochondria. CoQ10 is a lipid-soluble radical scavenger that helps keep the electron transport chain stable. Melatonin enhances GPX4 activity, reduces iron-catalyzed ROS production, and stabilises mitochondrial membranes. Clinical data indicate that both drugs improve oocyte competence and embryo development in IVF settings, likely by inhibiting ferroptosis-related lipid oxidation.

From a translational perspective, these molecular findings alter the perceptions of medical professionals regarding ovarian ageing. The ferroptosis framework posits that granulosa cell failure precedes oocyte failure, suggesting that the restoration of granulosa cell anti-ferroptotic capacity may preserve oocyte competence, even in women with low AMH or reduced follicle count, rather than viewing diminished ovarian reserve as an irreversible loss of oocytes. This paradigm posits that the ovary is not merely a passive victim of time, but an organ with biochemical systems capable of modification and response to targeted interventions. The clinical implications extend to IVF stimulation. Elevated mitochondrial ROS resulting from gonadotropin-induced steroidogenesis may surpass impaired *SIRT1* or Nrf2 pathways. To safeguard granulosa cells during COS, individuals with metabolic dysfunction, PCOS, or endometriosis-conditions marked by ferroptotic priming-may require tailored stimulation protocols alongside anti-ferroptotic interventions, including mitochondrial antioxidants, NAD^+^ boosters, and Nrf2 activators.

## 9. Discussion

Our narrative review synthesises evidence from cellular, transcriptomic, animal, and clinical studies, demonstrating that oxidative stress and ferroptosis are interconnected molecular processes that culminate in granulosa cell dysfunction, follicular atresia, and ovarian ageing. Increased ROS, iron overload, glutathione depletion, downregulation of GPX4, suppression of *SIRT1*-Nrf2 activity, mitochondrial collapse, and ferroptotic cell death are molecular signatures that consistently manifest across various models, including ageing ovaries, PCOS, reduced ovarian reserve, chemotherapy-induced injury, environmental toxicant exposure, and genetic anomalies. Our synthesis demonstrates that *SIRT1* and Nrf2 form the follicle’s primary anti-ferroptotic axis, safeguarding granulosa cells by preserving GPX4-dependent lipid peroxide detoxification, restoring mitochondrial homeostasis, and activating antioxidant transcriptional pathways. The literature reviewed indicates that numerous ovarian pathologies and ovarian ageing ought to be regarded as *SIRT1*-Nrf2 failure syndromes, wherein granulosa cells are driven towards ferroptotic death and oocyte quality is diminished due to a decreased resilience to oxidative damage.

### 9.1. Oxidative Stress as the Conserved Driver of Granulosa-Cell Dysfunction

According to our research, all studies examined oxidative stress as the most consistent and enduring molecular insult responsible for granulosa-cell malfunction, follicular atresia, and ovarian ageing. Granulosa cells undergo failure owing to a redox-dependent disturbance of cellular homeostasis induced by several upstream triggers, rather than a unique cause, despite each research analysing the issue from different physiological or pathological perspectives [210]. Liu et al. demonstrated that oxidative stress impairs PI3K-AKT, MAPK, FOXO, NF-κB, and Nrf2 signaling in granulosa cells, indicating that redox imbalance concurrently affects metabolic, apoptotic, inflammatory, and antioxidant pathways [31]. We now understand the molecular foundation by which ROS excess directly undermines cellular identity, due to their disruption of intracellular circuits. Yan et al. expanded the biological context by demonstrating that oxidative stress accelerates ovarian ageing at both the tissue and organismal levels via processes including telomere shortening, mitochondrial failure, apoptosis, and biomacromolecular damage [94]. Yan et al. emphasise lifestyle and environmental variables that produce ROS, thereby hastening follicular depletion, including ageing, smoking, high-sugar diets, pollution, and chemotherapeutics [11]. Liu et al. concentrate on granulosa cell signaling. These two investigations together demonstrate that oxidative stress is a biological and environmental component contributing to ovarian decline [31].

Gao et al. and Ghantabpour et al. support this perspective by asserting that the Nrf2/Keap1 system is crucial for the antioxidant defence of the ovaries [151,160]. Gao et al. contend that Nrf2 malfunction correlates with both pathological and physiological ovarian ageing, including reduced reproductive capability and systemic effects such as osteoporosis and cardiovascular disease [160]. Ghantabpour et al. describe oxidative stress as a “double-edged sword” essential for signaling but detrimental when mismanaged, thereby expanding the significance of Nrf2 to include all ovarian dysfunctions and infertility diseases [151]. In contrast to the results of Liu and Yan, these investigations demonstrate that oxidative stress becomes detrimental when antioxidant compensation is inadequate, as seen by the decrease of Nrf2, which signifies the transition from beneficial to harmful ROS signaling [11,31]. Akino et al. provided the most conclusive mechanistic evidence in human luteinized granulosa cells. The activation of Nrf2 by dimethyl fumarate increases the expression of catalase, SOD1, and OGG1, while concurrently reducing intracellular ROS and oxidative DNA damage [211]. This presents experimental proof of oxidative DNA damage and telomere erosion as mentioned by Yan et al. and Liu et al., while also affirming that Nrf2 safeguards cells [11,31]. Akino’s study demonstrates that granulosa cells may produce significant antioxidant responses when adequately stimulated [211]. This supports Gao’s finding that decreased Nrf2 signalling results in a lower capacity of the ovary to combat oxidative stress with advancing age [160].

Zhang et al. extended these results to an in vivo setting using a D-galactose-induced premature ovarian failure model, revealing that oxidative stress increases p16 and NLRP3 inflammasome components, while simultaneously decreasing levels of Nrf2, GCLC, HO-1, and NQO1 [212]. This illustrates that oxidative stress not only directly damages granulosa cells but also triggers inflammatory cascades that accelerate tissue ageing, similar to the inflammatory amplifications reported by Yan et al. Zhang et al. link redox imbalance to ovarian inflammatory ageing by demonstrating that oxidative damage across the body induces follicular senescence [94]. In contrast, Akino et al. examined the autonomic responses of granulosa cells [211]. Sharata et al. presented a therapeutically relevant toxicological viewpoint, demonstrating that cyclophosphamide-induced POF arises from oxidative stress and the activation of NF-κB/NLRP3 signaling, whereas the Nrf2/HO-1 pathway serves as a protective mechanism [213]. The pharmacological model and Zhang’s D-gal ageing model are analogous due to the predominance of oxidative stress over antioxidant defence, resulting in inflammation and the apoptosis of granulosa cells [212]. Sharata et al. demonstrate that many therapeutic compounds, including melatonin, berberine, curcumin, irbesartan, and resveratrol, function similarly by reinstating Nrf2 signaling [213]. Voros et al. include these metabolic pathways into the architecture of the follicular microenvironment [44]. The ovary is particularly susceptible to oxidative damage because to elevated amounts of PUFAs, iron-dependent metabolism, and steroidogenic activity, which generate ROS. Their story asserts that oxidative stress is not a mere accidental observation but an intrinsic feature of follicular physiology that becomes detrimental when not controlled by Nrf2 and *SIRT1*. This indicates that the ovary is inherently susceptible to oxidative injury and corroborates Yan’s discovery that lifestyle choices and environmental exposures elevate ROS [11]. Finally, Hu et al.’s GPX4 ageing model demonstrates that the downregulation of GPX4 and depletion of glutathione—precisely, the biochemical conditions requisite for ferroptotic lipid peroxidation occur with advancing age [214]. This demonstrates a clear correlation between oxidative stress and ferroptotic mechanisms.

### 9.2. Ferroptosis as the Unifying and Conserved Mode of Granulosa-Cell Death Across Ovarian Disorders

Ferroptosis is recognised as the final, universal mechanism of granulosa cell death in multiple conditions, such as PCOS, DOR, POI, chemotherapy-induced gonadotoxicity, exposure to environmental toxins, and age-related follicular decline. While apoptosis and necrosis have traditionally been the focus of reproductive biology, the accumulated evidence suggests that a ferroptotic pathway, based on GPX4 and SLC7A11, mostly dictates granulosa cell fate [100]. This axis is regulated by iron metabolism, lipid peroxidation, mitochondrial dysfunction, and antioxidant inadequacy. All investigations adhere to the same molecular sequence: iron accumulation → ROS accumulation → glutathione depletion → GPX4 failure → lipid peroxidation → mitochondrial contraction → ferroptotic cell death. This remains accurate despite the variation in stimuli.

Chen et al. provide a persuasive molecular elucidation by demonstrating that CTX triggers ferroptosis in granulosa cells by a synchronised overexpression of HO-1, reduction of GPX4, iron accumulation, and severe mitochondrial impairment [32]. Functional studies validated increased mitochondrial ROS, lipid peroxides, and mitochondrial depolarization indicative of ferroptosis while RNA sequencing analysis identified a ferroptosis enriched pathway in granulosa cells exposed to CTX. The suppression of HO-1 significantly decreased the depletion of GPX4, suggesting that CTX-induced redox stress directs granulosa cells towards ferroptosis rather than apoptosis [215]. This highlights HO-1 as a biomarker and regulator of ferroptotic vulnerability, establishing ferroptosis as pivotal to chemotherapy-induced ovarian failure, in alignment with Sharata et al.’s mechanistic framework of CP-induced oxidative damage.

Voros et al. asserted that the ovarian follicle’s high content of polyunsaturated fatty acids, reliance on iron-dependent steroidogenesis, and rich metabolic milieu creating reactive oxygen species make it intrinsically susceptible to ferroptosis [60]. Ferroptosis transpires in diverse clinically unrelated circumstances owing to the vulnerability of granulosa cells to lipid peroxidation, as dictated by the biochemical architecture of the follicle, according to their theoretical framework. Voros et al. underscore the significance of IVF results by identifying ferroptosis as a factor influencing reproductive success, therefore linking ferroptosis, cellular apoptosis, oocyte quality, and embryo viability [60].

Huang et al. expanded the ferroptosis framework to PCOS by finding differentially expressed genes linked to ferroptosis, which are enriched in ROS metabolism and mitochondrial membrane compartments [215]. Clinical validation demonstrates that these ferroptosis-associated genes have a favourable correlation with oocyte retrieval, MII maturity rate, fertilisation, and embryo quality. Huang et al. demonstrate that chronic metabolic and inflammatory abnormalities in PCOS modify granulosa-cell transcription to a ferroptotic phenotype, whereas Chen et al. concentrated on direct chemotoxicity [208,215]. This indicates that ferroptosis is a chronic maladaptive mechanism associated with metabolic reproductive problems, in addition to an acute toxic response. Yu et al. established that reduced ovarian reserve is associated with ferroptotic markers in granulosa cells, such as Prussian-blue-detectable iron accumulation, decreased expression of GPX4 and SLC7A11, mitochondrial shrinkage, and an increased apoptosis rate, in addition to the dysregulation of sterol biosynthesis and FSHR signalling [78]. DOR is defined by inherent ferroptotic susceptibility, shown by decreased FSH signalling and impaired aromatase activity, which impair mitochondrial antioxidant capacity and render granulosa cells prone to ferroptosis [216]. Conversely, PCOS (Huang et al.) is linked to an elevated oxidative-inflammatory load that is related to ferroptosis [208]. Yu et al.’s findings position ferroptosis as a crucial mechanistic link between follicular competence, mitochondrial integrity, and steroidogenesis [216].

Hu et al. (GPX4 ageing) conducted critical in vivo validation, demonstrating that aged ovaries display glutathione depletion, downregulation of GPX4, and ferroptotic mitochondrial morphology in granulosa cells [214]. In murine trials, ferroptosis inhibitors such as ferrostatin-1 shown enhancements in both the quantity and quality of oocytes, as well as preservation of follicular reserve. They also reversed traits previously considered irreversible consequences of ageing. The work by Hu et al. correlates age-related ovarian decline with ferroptotic pathways associated with PCOS and DOR, positing ferroptosis as a fundamental mechanism of ageing, as opposed to chemical toxicants (Chen et al.) or metabolic dysfunction (Huang et al.) [208,214,215].

Wang et al. advance the comprehension of ferroptosis by discovering a genetic variation of POI associated with BNC1 loss, in which NF2-YAP hyperactivation triggers oocyte ferroptosis [172]. Wang et al. demonstrate that oocytes are vulnerable to ferroptosis and that pharmacological reduction of YAP or blocking of ferroptosis may restore POI characteristics, in contrast to research focused on granulosa cells [172]. Their results validate the GPX4-aging data from Hu et al. and illustrate that ferroptosis directly influences oocytes instead of just impacting granulosa cells, thereby reinterpreting follicular atresia as a bidirectional ferroptotic process [214]. Insufficient levels of CSE1L result in excessive accumulation of FoxO1 inside the nucleus. This results in enhanced transcription of NCOA4, hence augmenting ferritinophagy, which subsequently enlarges the labile iron pool and initiates ferroptosis. Hu et al. identified a novel genetic mechanism of ferroptosis associated with the CSE1L mutation [214]. This mechanistic connection between nucleocytoplasmic transport and iron metabolism reveals a novel ferroptotic vulnerability route that is unaffected by redox stimuli. This categorises genetic POI with chemotherapy-induced damage, PCOS, DOR, and ageing in terms of mechanism. Geng et al. linked environmental toxicity to reproductive ferroptosis by demonstrating that fluoride exposure increases p53 activation, iron concentrations, and lipid peroxidation, while decreasing Nrf2, GPX4, and SLC7A11 levels [217]. Their research shows that extended environmental exposures may externally cause ferroptosis, linking toxicant-induced ovarian dysfunction with the ferroptotic markers identified in ageing, metabolic diseases, and CTX damage.

### 9.3. SIRT1-Nrf2 Crosstalk as the Central Anti-Ferroptotic Gatekeeper of Granulosa-Cell Fate

*SIRT1* and Nrf2 consistently emerge as the primary molecules safeguarding the viability of granulosa cells throughout all examined studies. They together safeguard the organism against oxidative stress, maintain mitochondrial equilibrium, regulate iron metabolism, and inhibit ferroptosis [178]. The research examines *SIRT1* and Nrf2 from several biological perspectives, including ageing, PCOS, chemotherapy-induced damage, premature ovarian insufficiency, metabolic disorders, and environmental toxicity. Nonetheless, they all adhere to the same molecular pattern: when *SIRT1*-Nrf2 signaling is functional, granulosa cells acclimatise to oxidative stress; when the axis malfunctions, ferroptosis occurs.

Liu et al. demonstrated that oxidative stress influences several antioxidant and survival pathways, including FOXO, PI3K-AKT, MAPK, NF-κB, and Nrf2 [31]. Their study indicates that Nrf2 is a major transcriptional centre influenced by redox imbalance. Yan et al. demonstrate that age-related oxidative stress in the ovaries concurrently diminishes the activities of *SIRT1* and Nrf2 [11]. This results in telomere erosion, apoptosis, inflammation, and mitochondrial damage alterations that increase the susceptibility of granulosa cells to ferroptosis. Yan et al. discuss the prolonged dysfunction of antioxidant networks, while Liu et al. address the immediate cessation of pathways [11]. Ghantabpour et al. expand the framework by establishing Nrf2 as a universal component for ovarian resiliency across various dysfunctions [151]. Gao et al. reinforce the pivotal function of Nrf2 by demonstrating that the disruption of the Nrf2/Keap1 pathway accelerates ovarian ageing [160]. Gao and Ghantabpour contend that the reduction of Nrf2 is a causative role in ovarian dysfunction, whereas Yan et al. describe the drop of Nrf2 as a result of ageing [151,160]. These data indicate that Nrf2 functions not only as an antioxidant transcription factor but also has a significant role in ovarian longevity.

Akino et al. provide clear mechanistic confirmation by demonstrating that the activation of Nrf2 in human granulosa cells with dimethyl fumarate elevates catalase, SOD1, and OGG1 levels while reducing reactive oxygen species and oxidative DNA damage [211]. This research demonstrates that the activation of Nrf2 in human reproductive cells is not only theoretical; it is effective. This corroborates the concepts proposed by Liu and Gao [31,160]. Nrf2 serves as a multifaceted defender within the reproductive system by concurrently regulating ROS detoxification, DNA repair, and redox homeostasis. Zhang et al. demonstrate that in a model of ageing and POF, D-galactose-induced ovarian failure is marked by a reduction in Nrf2, GCLC, NQO1, and HO-1, with an elevation in p16 and oxidative damage [218]. Zhang et al. demonstrate that Nrf2 is both essential and sufficient to rectify granulosa-cell malfunction, shown by the decline of Nrf2 levels in vivo and the reversal of ovarian ageing phenotypes with restoration of Nrf2 with daphnetin [218]. This differs from Akino et al., who only examined cultured human GCs [211].

Research focusing on *SIRT1* examines mitochondrial health, metabolic signalling, and redox adaptability reliant on deacetylase activity. Research centred on Nrf2 examines the regulation of transcriptional antioxidants. Alam et al. were the pioneers in identifying *SIRT1*, which they described as a universal regulator of reproduction and metabolism [122]. Their findings indicated that the loss of *SIRT1* elevates DNA damage, apoptosis, lipid peroxidation, and mitochondrial reactive oxygen species in both male and female gametes. Their study indicates that the ovary is more susceptible to oxidative and ferroptotic damage in the absence of sufficient *SIRT1*, a systemic antioxidant regulator. Li et al. elucidate the role of *SIRT1* by directly associating it with ovarian function [219]. Two *SIRT1* activators, calorie restriction and metabolic stabilisation, enhance ovarian reserve, stabilise granulosa cell activity, and improve reproductive outcomes associated with PCOS. Li et al. assert that *SIRT1* is present in the ovarian follicle and regulates the proliferation of granulosa cells, steroidogenesis, and the organism’s reaction to stress [219]. This differs from Alam et al., who examine the metabolic functions of the whole organism [122].

An et al. elucidate the most persuasive mechanistic link between ferroptosis and *SIRT1* [220]. Their study indicates that *SIRT1* has reduced activity in granulosa cells of individuals with PCOS. They discovered that the overexpression of *SIRT1* or its activation by pharmacological agents (SRT1720) inhibits ferroptosis, lipid peroxidation, reactive oxygen species accumulation, and GPX4 activity. *SIRT1* physically interacts with Nrf2 and promotes its deacetylation. This enhances Nrf2 activity in the nucleus and elevates the transcription of genes regulating iron and antioxidants. This is the clearest evidence that *SIRT1* functions upstream of Nrf2, activating it via post-translational modification rather than just being next to it. An et al. assert that the *SIRT1* → Nrf2 → GPX4 pathway constitutes the main anti-ferroptotic cascade in granulosa cells and may serve as a promising target for addressing infertility associated with PCOS [220].

Geng et al. assert that environmental contaminants such as fluoride enhance p53 activity, diminish the *SIRT1*-Nrf2-SLC7A11-GPX4 axis, increase iron levels and lipid peroxidation, and inhibit hormone secretion [217]. Their findings align with Chen et al.’s cyclophosphamide model, although they also indicate that *SIRT1*-Nrf2 suppression is a significant contributor to ovarian dysfunction induced by toxicants, beyond only pharmaceuticals [221].

### 9.4. From Molecular Mechanisms to Translational Opportunities in IVF and Fertility Preservation

The aggregate evidence from all analysed studies demonstrates that oxidative stress, ferroptosis, and the dysfunction of the *SIRT1*-Nrf2 axis are both mechanistic factors in granulosa-cell impairment and clinically significant pathways that directly affect IVF success, fertility preservation, and the management of ovarian ageing. Although each study investigates ovarian protection from distinct physiological, pathological, or pharmacological viewpoints, their findings converge, suggesting an emerging translational framework: restoring redox equilibrium and inhibiting ferroptosis may augment oocyte competence, conserve the ovarian reserve, and enhance assisted reproduction outcomes.

Liu et al. originally validated this notion by identifying resveratrol, sulforaphane, and other antioxidants as substances capable of mitigating oxidative damage to granulosa cells [31]. Their findings suggest that in vitro-modulated molecular pathways PI3K-AKT, FOXO, MAPK, and Nrf2-represent viable pharmaceutical targets for reproductive treatment. Yan et al. made considerable progress in this research by demonstrating that lifestyle choices and environmental exposures lead to persistent ROS buildup, hence contributing to ovarian ageing [11]. This indicates that antioxidant therapies may fulfil both preventative and therapeutic roles in reproductive medicine. Their paradigm advocates for the use of antioxidant, anti-inflammatory, and mitochondrial-targeted agents to alleviate ovarian ageing and improve ovarian reserve outcomes.

Gao et al. and Ghantabpour et al. significantly advanced the field by identifying Nrf2/Keap1 as the primary regulator that safeguards the ovaries [151,160]. Gao et al. showed that Nrf2 activation enhances antioxidant defences, reduces oxidative damage, and decelerates ovarian ageing [160]. Ghantabpour et al. subsequently used this concept to study several ovarian issues [151]. These investigations together suggest that targeting Nrf2 is not only beneficial but possibly essential for maintaining ovarian resilience, hence warranting therapeutic trials of Nrf2 activators in women facing reduced fertility or undergoing gonadotoxic therapy. Akino et al. demonstrated at the cellular level that pharmacological stimulation of Nrf2 reduces oxidative DNA damage in granulosa cells, stabilises catalase and SOD1, and preserves redox equilibrium [211]. Their data explicitly supports the integration of Nrf2-activating techniques into IVF preparation regimens, particularly for women with diminished antioxidant capacity, PCOS-related granulosa cell malfunction, or advanced reproductive age. Zhang et al. further validated this in vivo, showing that daphnetin restores ovarian function in models of premature ovarian failure via Nrf2 activation and NLRP3 inflammasome suppression. Their findings indicate that ovarian ageing is reversible and that pharmacological restoration of antioxidant pathways may enhance follicular function [212].

Sharata et al. established direct clinical relevance by demonstrating that several compounds—namely resveratrol, melatonin, quercetin, berberine, curcumin, and irbesartan—protect against cyclophosphamide-induced ovarian damage by modulating Nrf2/HO-1 and mitigating oxidative-inflammatory pathways [213]. Their summary identifies these drugs as possible adjuvants for fertility preservation techniques in cancer patients, integrating reproductive medicine with survivorship care. Sharata et al.’s model corroborates Chen et al.’s results that CTX causes ferroptosis in granulosa cells via GPX4 depletion, HO-1 dysregulation, and mitochondrial failure, underscoring the therapeutic potential of modulating HO-1 and inhibiting ferroptosis [213,221]. Voros et al. incorporated these molecular findings within the framework of IVF, showing that ferroptosis disrupts granulosa-cell steroidogenesis, reduces oocyte competence, and impedes optimum embryo development [44]. Their proposal to include ferroptosis indicators such as GPX4 levels, glutathione concentration, lipid ROS, and iron load into follicular-fluid diagnostic panels represents a significant advancement in conceptualisation. This aligns with the findings of Huang et al., which indicate that gene profiles associated with ferroptosis are intricately connected to oocyte retrieval, MII maturation rate, fertilisation, and embryo quality in PCOS [208]. These data suggest that ferroptosis profiling may develop into a new class of indicators for oocyte quality.

Yu et al. demonstrated that reduced ovarian reserve is characterised by iron buildup, mitochondrial ferroptosis, and the inhibition of GPX4 and SLC7A11, therefore linking sterol dysregulation and impaired FSHR signalling to redox susceptibility [216]. Their study suggests that treatment strategies designed to enhance FSHR signalling, restore GPX4 activity, or reduce iron buildup may mitigate granulosa cell degeneration linked to DOR. Hu et al. (GPX4 ageing) validated this therapeutic notion by demonstrating that ferroptosis inhibitors improve oocyte quantity and quality in aged mice and ameliorate CTX-induced ovarian damage [214]. Their findings clearly refute the longstanding belief that ovarian decline is permanent, facilitating the development of “ovarian rejuvenation” techniques focused on ferroptosis control. Geng et al. included environmental toxicity into the translational dialogue by demonstrating that fluoride suppresses Nrf2, GPX4, and SLC7A11, while augmenting p53 activity, iron buildup, and lipid peroxidation [217]. Their results underscore the necessity for environmental risk assessment in reproductive health and suggest that antioxidant and anti-ferroptotic therapy may benefit women exposed to chronic environmental toxicants.

## 10. Clinical Implications

Our review shows that oxidative stress and ferroptosis are two important methods that doctors can use to treat problems with granulosa cells, ovarian ageing, and bad IVF results. Ageing, PCOS, DOR, chemotherapy-induced POI, and environmental exposures consistently reduce *SIRT1*, Nrf2, GPX4, and glutathione metabolism. This suggests that these molecular disruptions are not unique to specific diseases but are common therapeutic targets. This framework assists reproductive health professionals in incorporating mitochondrial-supporting, anti-ferroptotic, and antioxidant therapies into individualised treatment regimens. Resveratrol, melatonin, daphnetin, NAD^+^ boosters, and Nrf2 activators are some of the agents that show promise for restoring redox balance and improving follicular function. Ferroptosis inhibitors may be particularly beneficial for women undergoing gonadotoxic chemotherapy or possessing a diminished ovarian reserve. Biomarkers related to ferroptosis, like GPX4, SLC7A11, lipid peroxides, and follicular-fluid iron, have been linked to the ability of oocytes to function. This suggests that biomarker-guided IVF protocols could improve the prediction of ovarian response and embryonic developmental potential. Genetic susceptibilities associated with BNC1, CSE1L, or other genes that regulate ferroptosis may facilitate precision fertility medicine. When looked at together, these results suggest that changing the *SIRT1*-Nrf2-GPX4 axis in a therapeutic way may help keep the ovaries working, improve the results of IVF, and slow down reproductive ageing. This is a way to help women have better reproductive health based on how it works.

Integrating ferroptosis regulation, *SIRT1*/Nrf2 activation, and antioxidant-based therapies into normal clinical practice remains problematic, despite strong mechanistic justification from experimental investigations. The majority of the current information derives from research conducted on animals or in vitro granulosa cell models. These may not precisely represent the complexity, variability, and hormonal regulation of the human ovarian follicle. Currently, human clinical data is scarce, sometimes indirect, and usually complicated due to patient heterogeneity, stimulation regimens, metabolic condition, and age-related variability. Furthermore, redox signalling has a physiological function in steroidogenesis and ovulation, indicating that indiscriminate antioxidant or route manipulation might interfere with critical reproductive processes. Therefore, before the actual use of these techniques, rigorously planned prospective clinical trials are necessary to determine safety, timing, dose, and criteria for patient selection.

## 11. Conclusions

Oxidative stress, ferroptosis, and the disruption of the SIRT1-Nrf2 defence axis influence granulosa cell dysfunction, follicular atresia, and ovarian ageing. Emerging experimental and translational evidence indicates that iron accumulation, glutathione depletion, GPX4 inhibition, unchecked lipid peroxidation, and mitochondrial dysfunction collaboratively contribute to the demise of granulosa cells across various pathological contexts, including polycystic ovary syndrome, diminished ovarian reserve, primary ovarian insufficiency, gonadotoxic chemotherapy, environmental toxin exposure, and chronological ageing. In this context, Nrf2 and SIRT1 collaborate to inhibit ferroptosis: Nrf2 maintains redox homeostasis by regulating the transcription of antioxidant enzymes, glutathione synthesis, and iron-sequestering pathways, whereas SIRT1 enhances mitochondrial integrity and metabolic stress responses, in part by activating Nrf2. Granulosa cells may withstand oxidative stress while this axis is intact; however, when disturbed, cellular destiny transitions to ferroptosis, compromising oocyte competence and hastening ovarian decline. Insufficient direct clinical evidence exists in people when attempting to connect ferroptosis and SIRT1-Nrf2 dysregulation to ovarian ageing, despite robust molecular confirmation from in vitro and animal research. The variety of granulosa cells, inter-individual variability, and variations in ovarian stimulation regimens complicate the application of this to real-world scenarios. Furthermore, indiscriminate modification of oxidative pathways may have unforeseen consequences, since redox signalling is crucial for folliculogenesis and ovulation. The lack of standardised ferroptosis-related biomarkers relevant to reproductive organs and the scarcity of longitudinal human data further restrict current study. Future research should focus on integrated experimental approaches that include human granulosa cells, studies of follicular fluid, and relevant animal models to clarify the temporal and causal role of ferroptosis in follicular decline. The discovery and confirmation of biomarkers related to ferroptosis, including lipid peroxidation products, iron-handling proteins, and redox-regulated RNAs, might enhance the translational classification of ovarian ageing phenotypes. Prior to extensive clinical use, carefully structured pilot clinical studies assessing targeted redox-modulating strategies emphasizing timing, dose, and patient selection are crucial to determine feasibility and safety.

## Figures and Tables

**Table 1:** Core Molecular Mechanisms of Ferroptosis in Human Granulosa Cells.

Molecular Axis	Key Molecules	Mechanistic Role in Ferroptosis	Observed Effects in Granulosa Cells
Iron Import & Accumulation	TFRC, DMT1	Increased uptake of Fe^2+^ promotes Fenton chemistry and ROS amplification.	Elevated labile iron pool, enhanced lipid-ROS, iron-dependent mitochondrial damage.
Ferritinophagy/Iron Release	NCOA4, FTH1, FTL	Autophagic degradation of ferritin releases redox-active iron.	Iron overload, sensitivity to oxidative injury, disruption of steroidogenesis.
Lipid Peroxidation Initiation	ACSL4, LPCAT3	Incorporation of PUFA-CoA into phospholipids, priming membranes for oxidation.	High PUFA-PL content, membrane vulnerability to peroxidation.
Lipid Peroxidation Propagation	ALOX12/15, POR	Enzymatic peroxidation of PUFA-PL, uncontrolled ROS amplification.	Accumulation of lipid peroxides, mitochondrial shrinkage, cytoskeletal collapse.
Antioxidant Failure (GSH Depletion)	SLC7A11/xCT, GCLC/GCLM	Impaired cystine import and impaired glutathione synthesis weaken redox buffering capacity.	Collapse of redox homeostasis, accumulation of lipophilic radicals.
GPX4 Inactivation	GPX4 protein, Se-dependent pathways	Loss of detoxification of lipid hydroperoxides leads to catastrophic membrane damage.	Hallmark ferroptotic phenotype: condensed mitochondria, cristae disappearance.
Mitochondrial Dysfunction	DRP1, OPA1, MFN1/2	Disrupted dynamics intensify ROS production and inhibit electron transport chain.	Depolarized membranes, mtROS accumulation, bioenergetic failure.
Cell-Death Execution	Lipid ROS, iron-driven radicals	Terminal peroxidation of membranes leads to regulated necrotic death.	Granulosa-cell loss, impaired oocyte support, follicular atresia.

A summary of the primary molecular mechanisms governing ferroptosis in human granulosa cells. Ferroptosis results from excessive iron accumulation, phospholipid peroxidation, diminished GSH levels, and the inactivation of GPX4. The distinctive ultrastructural feature of mitochondrial shrinkage accompanied by the loss of cristae signifies the last phase of ferroptotic cell death. These methods together render the follicles less stable, impair the functionality of the oocytes, and diminish the viability of the granulosa cells.

**Table 2:** Principal Molecular Functions of *SIRT1* in Human Granulosa Cells.

Functional Axis	Molecular Targets/Pathways	Mechanistic Effects of *SIRT1*	Consequences in Granulosa Cells
Mitochondrial Biogenesis & Dynamics	PGC-1α, TFAM, OPA1, MFN1/2, DRP1	Deacetylates PGC-1α, promotes mitochondrial biogenesis, enhances fusion, limits excessive fission.	Improved ATP production, stabilized membrane potential, reduced mtROS, greater oocyte support.
Antioxidant & Redox Defense	FOXO3, SOD2, Catalase, Nrf2	Activates FOXO3, increases antioxidant gene transcription, deacetylates Nrf2 to enhance nuclear retention.	Reduced ROS load, enhanced resistance to oxidative injury, prevention of GSH depletion.
Anti-Ferroptotic Protection	GPX4, SLC7A11, GSH synthesis pathways	Stabilizes GPX4 expression, promotes cystine/GSH availability, restrains lipid peroxidation.	Suppression of lipid-ROS accumulation; protection against ferroptotic cell death.
Mitophagy & Mitochondrial Quality Control	PINK1–Parkin axis	Activates mitophagy, clears damaged mitochondria.	Reduced dysfunctional mitochondria, enhanced mitochondrial turnover, protection from apoptosis/ferroptosis.
Inflammation & Stress Signalling	NF-κB, MAPK, JNK, p53	Deacetylates NF-κB subunits, restrains inflammatory cascades; modulates p53 activity.	Reduced inflammatory signalling, decreased cytokine-induced ROS, improved cell survival.
Steroidogenic Regulation	CYP19A1 (Aromatase), StAR, FSHR/CREB pathway	Enhances steroidogenic gene expression via deacetylation-dependent coactivator control.	Improved estradiol synthesis, enhanced FSH responsiveness, better oocyte competence.
Metabolic Homeostasis & NAD^+^ Economy	NAMPT–NAD^+^ salvage pathway, AMPK, mTOR	Links energy status to transcriptional programs; increases NAD^+^ availability for sirtuin signalling.	Higher metabolic resilience, reduced lipotoxic stress, improved follicular survival.
Aging-Related Protection	Telomere regulators (TRF2, TERT), SASP markers	Suppresses senescence pathways and SASP phenotype via FOXO3 and NF-κB control.	Delayed GC aging, preserved follicular reserve, reduced atresia.

A synopsis of the primary molecular functions of *SIRT1* in human granulosa cells. *SIRT1* regulates the PGC-1α, FOXO3, Nrf2, GPX4, and mitophagy pathways to bring together metabolic signals, redox balance, mitochondrial health, and ferroptotic suppression. *SIRT1* maintains granulosa cell viability, endocrine function, and oocyte maturation support through these mechanisms, especially in the context of oxidative or metabolic stress.

**Table 3:** Major Nrf2-Regulated Antioxidant and Anti-Ferroptotic Targets in Granulosa Cells.

Target/Pathway	Gene/Protein	Primary Function	Relevance to Granulosa Cells and Ferroptosis
Glutathione Biosynthesis	GCLC, GCLM	Catalytic and modifier subunits of glutamate–cysteine ligase, rate-limiting enzyme for GSH synthesis.	Maintain intracellular GSH pools required for detoxification of ROS and lipid peroxides; depletion sensitises granulosa cells to ferroptosis.
Heme and Iron Detoxification	HO-1 (HMOX1)	Degrades heme into biliverdin, CO and free iron; cytoprotective in controlled activation.	Modulates heme-derived oxidative stress; dysregulated or excessive HO-1 activity may contribute to iron overload and ferroptotic susceptibility.
Redox Cycling & ROS Neutralisation	NQO1, PRDXs, Trx/TrxR system	Support two-electron reduction of quinones and peroxides; limit redox cycling and oxidative damage.	Reduce oxidative burden in granulosa cells and protect mitochondrial and nuclear components from ROS-mediated injury.
Superoxide and Peroxide Detoxification	SOD1, SOD2, Catalase	Convert superoxide to H_2_O_2_ (SODs) and decompose H_2_O_2_ to water and oxygen (catalase).	Control cytosolic and mitochondrial ROS levels; reduced activity promotes mtROS accumulation and primes ferroptotic pathways.
Iron Storage and Sequestration	FTH1, FTL, Ferritin complex	Sequester excess iron in a redox-inert form, limiting Fenton chemistry.	Restrict expansion of the labile iron pool and prevent iron-driven lipid peroxidation in granulosa cells.
Cystine Uptake and GSH Maintenance	SLC7A11 (xCT)	Imports cystine in exchange for glutamate; precursor for GSH synthesis.	Supports glutathione-dependent GPX4 activity; downregulation is a key initiating event in ferroptotic cell death.
DNA Repair and Genomic Stability	OGG1, other BER components	Repair oxidative DNA lesions, particularly 8-oxoG.	Protects granulosa-cell genome from ROS-induced mutations and contributes to long-term follicular integrity.
Inflammation–Redox Interface	TXNIP, NLRP3 (indirect regulation)	TXNIP links oxidative stress to inflammasome activation; NLRP3 forms inflammatory complexes.	Dysregulated Nrf2 signalling permits TXNIP and NLRP3 upregulation, promoting inflammatory amplification of oxidative and ferroptotic damage.
Mitochondrial Function and Bioenergetics	NRF1/2, downstream respiratory-chain components (indirect)	Support mitochondrial transcription and respiratory capacity.	Nrf2-driven mitochondrial support helps maintain membrane potential and reduces mtROS, indirectly limiting ferroptosis.

Key Nrf2 downstream targets essential for the regulation of granulosa cell ferroptosis, iron metabolism, glutathione metabolism, and antioxidant defence. Nrf2 maintains redox homeostasis and constrains the labile iron pool by enhancing the synthesis of glutathione (GCLC/GCLM), promoting ROS-detoxifying enzymes (NQO1, SODs, catalase), and facilitating iron-sequestration proteins (FTH1/FTL). Conversely, when Nrf2 signalling is compromised, granulosa cells exhibit increased susceptibility to ferroptotic death resulting from cystine deficiency (SLC7A11 downregulation), GSH depletion, GPX4 inactivation, and inflammatory enhancement through TXNIP–NLRP3.

## Data Availability

No new data were created or analysed in this study. Data sharing is not applicable to this article.
